# *MutL homolog 1* participates in interference-sensitive meiotic crossover formation in soybean

**DOI:** 10.1093/plphys/kiae165

**Published:** 2024-03-16

**Authors:** Tao Wu, Suxin Yang, Junling Fang, Yongheng Ye, Yaohua Zhang, Jinshan Gao, Jiantian Leng, Zhirui Zhang, Kuanqiang Tang, Javaid Akhter Bhat, Xianzhong Feng

**Affiliations:** Key Laboratory of Soybean Molecular Design Breeding, Northeast Institute of Geography and Agroecology, Chinese Academy of Sciences, Changchun 130102, China; College of Advanced Agricultural Sciences, University of Chinese Academy of Sciences, Beijing 100049, China; Key Laboratory of Soybean Molecular Design Breeding, Northeast Institute of Geography and Agroecology, Chinese Academy of Sciences, Changchun 130102, China; College of Advanced Agricultural Sciences, University of Chinese Academy of Sciences, Beijing 100049, China; Key Laboratory of Soybean Molecular Design Breeding, Northeast Institute of Geography and Agroecology, Chinese Academy of Sciences, Changchun 130102, China; College of Life Science, Jilin Agricultural University, Changchun 130118, China; Key Laboratory of Soybean Molecular Design Breeding, Northeast Institute of Geography and Agroecology, Chinese Academy of Sciences, Changchun 130102, China; College of Advanced Agricultural Sciences, University of Chinese Academy of Sciences, Beijing 100049, China; Key Laboratory of Soybean Molecular Design Breeding, Northeast Institute of Geography and Agroecology, Chinese Academy of Sciences, Changchun 130102, China; Key Laboratory of Soybean Molecular Design Breeding, Northeast Institute of Geography and Agroecology, Chinese Academy of Sciences, Changchun 130102, China; Key Laboratory of Soybean Molecular Design Breeding, Northeast Institute of Geography and Agroecology, Chinese Academy of Sciences, Changchun 130102, China; Key Laboratory of Soybean Molecular Design Breeding, Northeast Institute of Geography and Agroecology, Chinese Academy of Sciences, Changchun 130102, China; College of Advanced Agricultural Sciences, University of Chinese Academy of Sciences, Beijing 100049, China; Key Laboratory of Soybean Molecular Design Breeding, Northeast Institute of Geography and Agroecology, Chinese Academy of Sciences, Changchun 130102, China; Zhejiang Lab, Hangzhou 311121, China; Key Laboratory of Soybean Molecular Design Breeding, Northeast Institute of Geography and Agroecology, Chinese Academy of Sciences, Changchun 130102, China; Zhejiang Lab, Hangzhou 311121, China

## Abstract

MutL homolog 1 (MLH1), a member of the MutL homolog family, is required for normal recombination in most organisms. However, its role in soybean (*Glycine max*) remains unclear to date. Here, we characterized the *Glycine max female and male sterility 1* (*Gmfms1*) mutation that reduces pollen grain viability and increases embryo sac abortion in soybean. Map-based cloning revealed that the causal gene of *Gmfms1* is *Glycine max MutL homolog 1* (*GmMLH1*), and CRISPR/Cas9 knockout approach further validated that disruption of *GmMLH1* confers the female–male sterility phenotype in soybean. Loss of *GmMLH1* function disrupted bivalent formation, leading to univalent mis-segregation during meiosis and ultimately to female–male sterility. The *Gmmlh1* mutant showed about a 78.16% decrease in meiotic crossover frequency compared to the wild type. The residual chiasmata followed a Poisson distribution, suggesting that interference-sensitive crossover formation was affected in the *Gmmlh1* mutant. Furthermore, GmMLH1 could interact with GmMLH3A and GmMLH3B both in vivo and in vitro. Overall, our work demonstrates that GmMLH1 participates in interference-sensitive crossover formation in soybean, and provides additional information about the conserved functions of *MLH1* across plant species.

## Teaser

MutL homolog 1 forms a complex with MutL homolog 3 to regulate interference-sensitive crossover formation and affects female–male fertility in soybean.

## Introduction

Meiosis is a key process in the sexual reproduction of eukaryotes, involving two adjacent meiotic chromosome divisions (Meiosis Ⅰ and Meiosis Ⅱ) and generating 4 haploid spores from a single diploid parental cell (Mercier et al. 2015). The meiotic process is important for maintaining a constant number of chromosomes generation after generation. During Meiosis Ⅰ, the homologous chromosomes segregate, while in Meiosis Ⅱ, the sister chromatids of each chromosome segregate. In Prophase Ⅰ, homologous chromosomes pair via the formation of the synaptonemal complex. During this process, the homologous chromosomes are tethered together by crossovers (COs), which correspond to homologous recombination sites between homologs (Gray and Cohen 2016). Most eukaryotes possess two types of COs: Class I COs (interference-sensitive) and Class Ⅱ COs (interference-insensitive). The Class I COs are the ZMM (the protein complex is comprised of Zipper 1–4, MutS homolog 4–5, and meiotic recombination 3)-dependent interference-sensitive COs, while Class II COs are MUS81 (methyl methansulfonate, UV sensitive 81)-dependent and noninterfering (Börner et al. 2004). The ZMM complex asymmetrically resolves the double Holliday junctions (dHjs), yielding interference-sensitive Class Ⅰ COs, which represent 85% to 90% of total CO events. Class Ⅱ COs are generated by structure-speciﬁc endonuclease MUS81 (Higgins et al. 2008), and AtFANCD2 (*Arabidopsis thaliana* Fanconi Anemia Complementation Group D2) has been reported to be involved in the formation of Class Ⅱ COs in Arabidopsis (*A. thaliana*) (Kurzbauer et al. 2018). The formation of COs ensures the proper division of homologous chromosomes at Anaphase Ⅰ and plays a critical role during meiotic recombination.

COs are initiated by programmed DNA double-strand breaks, then followed by the generation of 3′-overhanging single-strand DNA, the displacement loop (D-loop), and dHjs, finally yielding two types of COs (Mercier et al. 2015; Wang and Copenhaver 2018). Most dHjs are cleaved into Class I COs by the MutLγ heterodimer (MLH1–MLH3), which is composed of MLH1 and MutL homolog 3 (MLH3) directed and stimulated by the component of ZMM complex MutSγ (MSH4–MSH5) (Cannavo et al. 2020). MLH1, a homolog of the bacteria MutL protein, is required for meiotic CO formation in yeast (*Saccharomyces cerevisiae*), mammals, and plants (Prolla et al. 1994; Dion et al. 2007; Lhuissier et al. 2007; Mao et al. 2021). The *AtMLH1* gene is highly expressed in young tissues during both vegetative and reproductive stages, and its mutation leads to reproductive defects in homozygotes (Jean et al. 1999; Dion et al. 2007). Mutations in MLH1-related protein reduced CO formation by approximately 60% in Arabidopsis (Jackson et al. 2006) and by 40% to 70% in rice (*Oryza sativa*) (Xin et al. 2021), leading to a seed-setting rate of approximately 20% in *Atmlh1* (Dion et al. 2007) and 14% in *Osmlh1* (Mao et al. 2021; Xin et al. 2021). In rice, OsMLH1 interacts with OsMLH3 to regulate synapsis, promoting Class I CO formation in the macrospore and microspore mother cells during meiosis, and the frequency of residual chiasmata was 47% to 72% in *Osmlh1* mutant (Mao et al. 2021; Xin et al. 2021).

Soybean (*Glycine max*) is an economically important crop that is grown worldwide for its seeds, which contain high levels of oil (approximately 20%) and protein (approximately 40%) (Zhang et al. 2018). Although the soybean genome has been sequenced, the molecular basis of soybean developmental traits remains largely unknown (Schmutz et al. 2010; Zhang et al. 2022). Recently, several genes involved in meiotic recombination have been identified across various crops, including rice, maize (*Zea mays*), wheat (*Triticum turgidum*), barley (*Hordeum vulgare*), lettuce (*Lactuca sativa*), and rapeseed (*Brassica napus*), providing the theoretical foundations for molecular breeding programs (Colas et al. 2016; Fayos et al. 2019; Gonzalo et al. 2019; Draeger et al. 2020; Jing et al. 2020; Li et al. 2021). Meiotic studies in soybean have spanned decades, and include research on classical asynaptic and desynaptic mutants (Owen 1928; Palmer 1974; Palmer and Horner 2000; Kato and Palmer 2003; Palmer et al. 2008; Speth et al. 2015; Baumbach et al. 2016). These findings provide detailed descriptions of the meiotic process and resources for research in soybean. However, meiotic studies at the molecular level are rare in soybean. In the present study, we characterized the *GmMLH1* homolog in soybean and investigated its roles in meiosis. Our findings reveal that *GmMLH1* participates in the formation of most COs and is essential for ensuring female and male fertility in soybean.

## Results

### Characterization of the *Gmfms1* mutant

**Figure 1.**
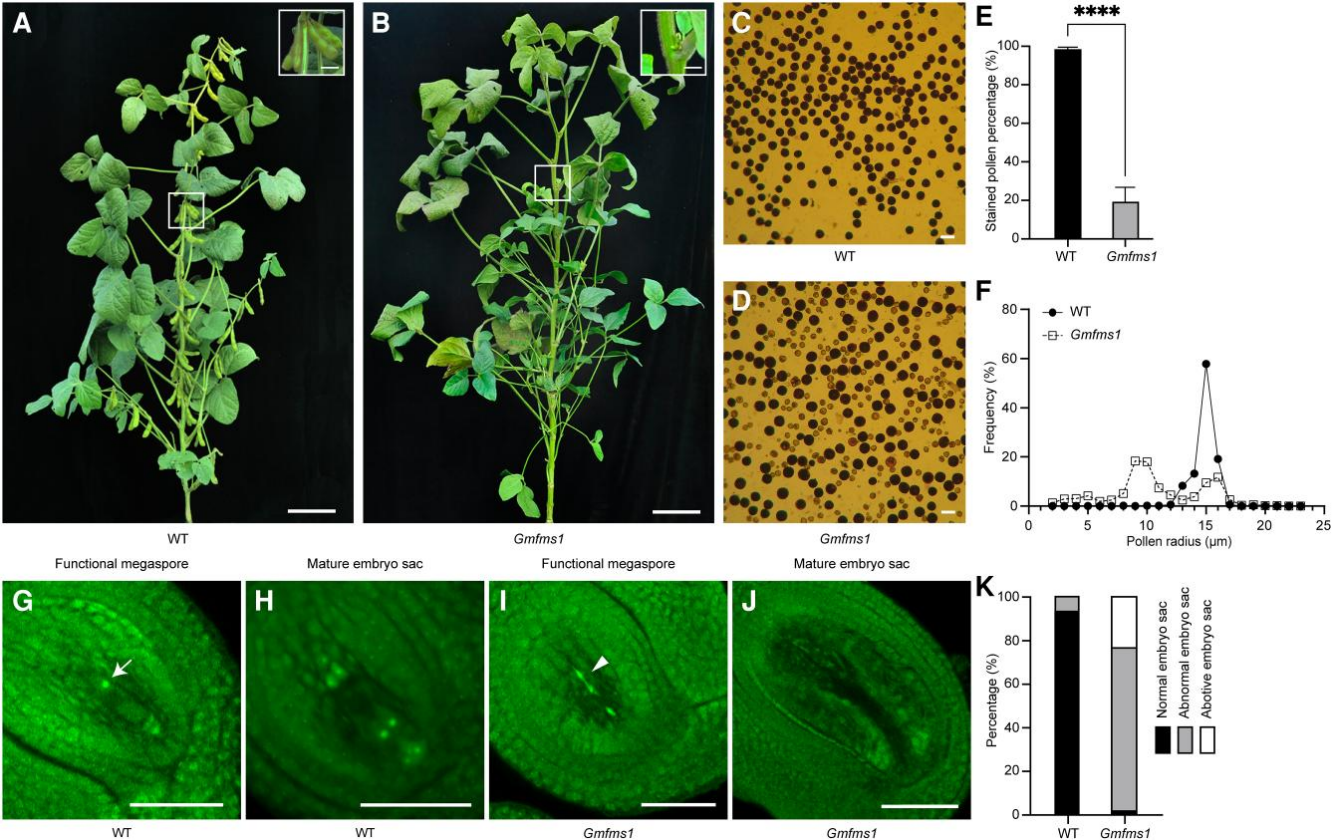
Phenotypic characterization of the *Gmfms1* mutant. **A)** WT plant and **B)***Gmfms1* mutant plant at the R6 stage, where the inserted images show the areas in white boxes at 2 × magnification. Scale bars = 10 cm. Scale bars (top-right boxes) = 2 cm. I_2_-KI staining of pollen grains of the wild type **C)** and *Gmfms1***D)**. Scale bars = 50 µm. **E)** Comparison of the stained pollen ratio between wild type (*n* = 4,237) and *Gmfms1* (*n* = 4,364); data shown are means ± SD of 3 replicate plants (****P < 0.0001, Student's unpaired *t*-test). **F)** Pollen grain size frequencies of the wild type (*n* = 524) and the *Gmfms1* (*n* = 624). **G, H)** Development of functional megaspore **G, I)** and mature embryo sac **H, J)** in the wild type **G, H)** and the *Gmfms1* mutant **I, J)**. Arrows indicate nuclei and arrowheads indicate degenerated nuclei in the functional megaspore. Scale bars = 50 µm. **K)** Quantification of the embryo sac fertility in the wild type (*n* = 63) and *Gmfms1* (*n* = 55). Abnormal embryo sacs are defined as those with polar nuclei of abnormal number or in abnormal positions. Abortive embryo sacs are defined as embryo sacs lacking any cellular structure.

The *Gmfms1* (*Glycine max female and male sterility 1*) mutant was identified from a diverse mutant population developed from the Williams 82 soybean cultivar in our laboratory, as previously described (Feng et al. 2019; Tang et al. 2020). Compared with the wild type, the mutant exhibited an obvious severe reduction in fertility (Fig. 1A and B). Iodine-potassium iodide (I_2_-KI) staining revealed reduced fertility of pollen grains in the mutant (Fig. 1C and D). A total of 80.66% of all mutant pollen grains were rarely stained by 1% I_2_-KI solution, appearing empty and shrunken (Fig. 1E). In addition, most of the mutant pollen grains were smaller in size compared to those of the wild type (Fig. 1F). To investigate female fertility in the mutant, functional megaspores and mature embryo sacs from the wild type and the sterile mutant were examined using the whole-mount stain-clearing laser scanning confocal microscopy (WCLSM). The embryo sacs developed normally in the wild type, with the classic 7-cell and 8-nuclei structure (Fig. 1G and H). However, in the mutant, the chalazal megaspores and micropylar megaspores degenerated, resulting in embryo sac abnormality and abortion (Fig. 1I and J). Few abnormal embryo sacs were observed in the wild type (6.35%), but high frequencies of aborted embryo sacs (22.92%) and abnormal embryo sacs (75.00%) were found in the sterile mutant (Fig. 1K). To further investigate the sterility phenotype in the mutant, we performed reciprocal cross tests between the mutant and the wild type. When the stigmas of the mutant were pollinated with wild-type pollen grains, the pod-setting rate was only 1.27% (*n* = 263), while, the pod-setting rate was 14.29% (*n* = 42) when the wild-type stigmas were pollinated with the pollen grains of the mutant. However, the pod-setting rate of wild-type stigmas and pollen grains was 53.66% (*n* = 41) in the same environment (Supplementary Table S1), indicating that the fertilities of both female and male were severely reduced. Based on these results, the defective development of both the pollen grains and the embryo sacs was closely associated with the serious reduction in fertility of the *Gmfms1* mutant plant.

### Isolation and cloning of the *GmMLH1* gene

To explore the molecular mechanisms underlying the gamete abnormalities observed in the *Gmfms1* mutant, an F_2_ population was generated by crossing heterozygous *GmFMS1*/*Gmfms1* plants with another elite Chinese soybean cultivar, Hedou 12. At the full maturity stage in the F_2_ population, plants with fewer than 2 seeds were regarded as mutants, while the others were considered to be wild type. Compared to the mature wild-type plants grown in the field, the mutant plants produced almost no seeds per plant (397.61 ± 134.16 in the wild type vs. 0.33 ± 0.49 in the mutant, *P* < 0.0001, Student's unpaired *t*-test) and an extremely small number of one-seeded pods per plant (25.51 ± 15.35 in the wild type vs. 0.33 ± 0.49 in the mutant, *P* < 0.0001, Student's unpaired *t*-test) (Supplementary Fig. S1C–F). Phenotypic analysis also indicated that there were no significant differences in plant height, branch number and node number between the wild type and mutant before harvest (Supplementary Fig. S1A, B and G–I). These results demonstrated that the *Gmfms1* mutant exhibited severely reduced fertility compared to the wild type in the field.

**Figure 2.**
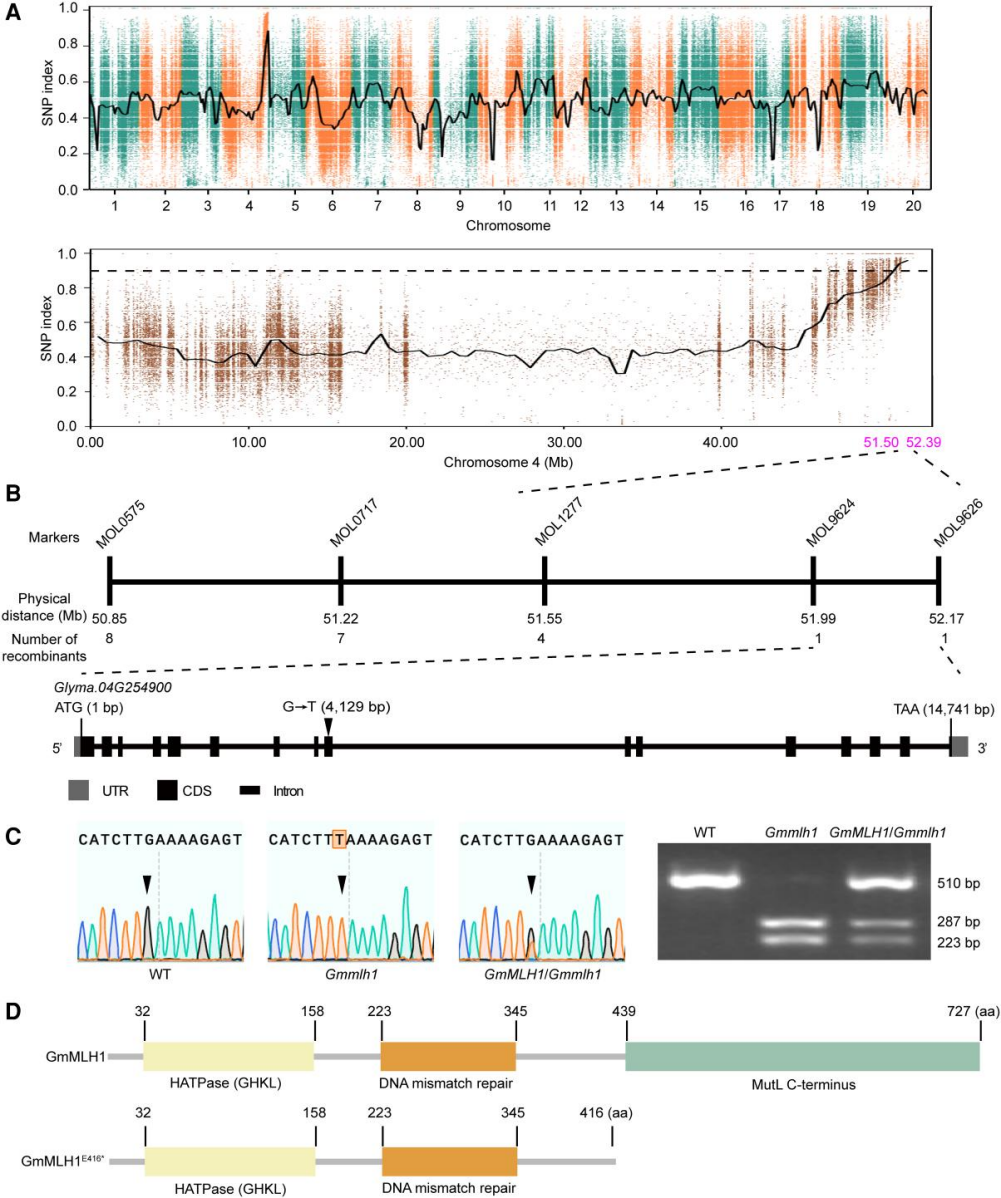
Mapping of the *GmMLH1* gene. **A)** SNP index plot covering all 20 chromosomes (upper) and for Chromosome 4 (lower) in the *Gmmlh1* mutant pool. The candidate region was identified between 50.31 and 52.15 Mb (shown in magenta font) of Chromosome 4 through BSA-seq. **B)** Diagram of the fine mapping of the *GmMLH1* gene with designed markers (upper), and the structure of the *Glyma.04G254900* gene (lower). A G-to-A substitution at 4,129 bp was detected in *Glyma.04G254900* in the *Gmmlh1* mutant. **C)** Characterization of the mutation of the *GmMLH1* gene. Sequence analysis of the *Glyma.04G254900* mutation site in the wild type, *Gmmlh1* homozygous mutant, and *GmMLH1*/*Gmmlh1* heterozygous plant. Genotype analysis using CAPS marker for the wild type, *Gmmlh1* homozygous mutant, and *GmMLH1*/*Gmmlh1* heterozygous plant. **D)** Structures of the wild-type GmMLH1 and mutant protein (GmMLH1^E416*^) in *Gmmlh1*. The GmMLH1^E416*^ protein is truncated due to a premature stop codon resulting from the substitution mutation in the *Gmmlh1* mutant. The boxes from left to right demarcate HATPase (GHKL) domains, DNA mismatch repair domains, and MutL C-terminus domain, respectively.

In this segregated F_2_ population, the segregation ratio of fertile to sterile was 3:1 (fertile, 142; sterile, 39; Chi-squared test, χ^2^ = 1.15 < 3.84; *P* > 0.05), indicating that *Gmfms1* mutant phenotype was governed by a single recessive mutation. Bulked segregant analysis-sequencing (BSA-seq) of the 39 sterile plants in the F_2_ population identified 2,358,586 single nucleotide polymorphisms (SNPs) and 371,109 small insertion and deletions (InDels). Based on differences in allele frequency, the *Gmfms1* locus was mapped in the physical interval of 51.50 to 52.39 Mb (0.89 Mb) on Chromosome 4 (Fig. 2A). Using the 937 F_2:3_ individuals and primers (MOL0575, MOL0717, MOL1277, MOL9346, MOL9624, and MOL9626) designed within the 51.50 to 52.39 Mb interval on Chromosome 4, the *Gmfms1* locus was further pinpointed to a 183-kb region between the MOL9624 (51.99 Mb) and MOL9626 (52.17 Mb) markers. This 183-kb region includes 21 annotated genes in the Williams 82 reference genome (*Glycine max Wm82.a2.v1*) (Fig. 2B). To screen the possible candidate gene causing the *Gmfms1* mutant phenotype from these 21 genes, we compared the genomic sequences of this entire 183 kb region between the wild-type and mutant soybean genotypes. We identified only a single nucleotide substitution: G to T at position 4,129 of *Glyma.04G254900* gene (Fig. 2B). Cleaved amplified polymorphism sequences (CAPS) genotype analysis confirmed that the G-to-T substitution in *Glyma.04G254900* cosegregated with the sterility phenotype in all *Gmfms1* mutants (Fig. 2C). This mutation in *Gmfms1* created a stop codon in the 9th exon of *Glyma.04G254900*, which resulted in the formation of a truncated protein (416 aa) compared to the wild type (727 aa) (Fig. 2D). Moreover, *Glyma.04G254900* was highly similar to the *AtMLH1* in Arabidopsis. Our results suggested that this mutation in *Glyma.04G254900* might be responsible for the mutant phenotype of *Gmfms1*.

**Figure 3.**
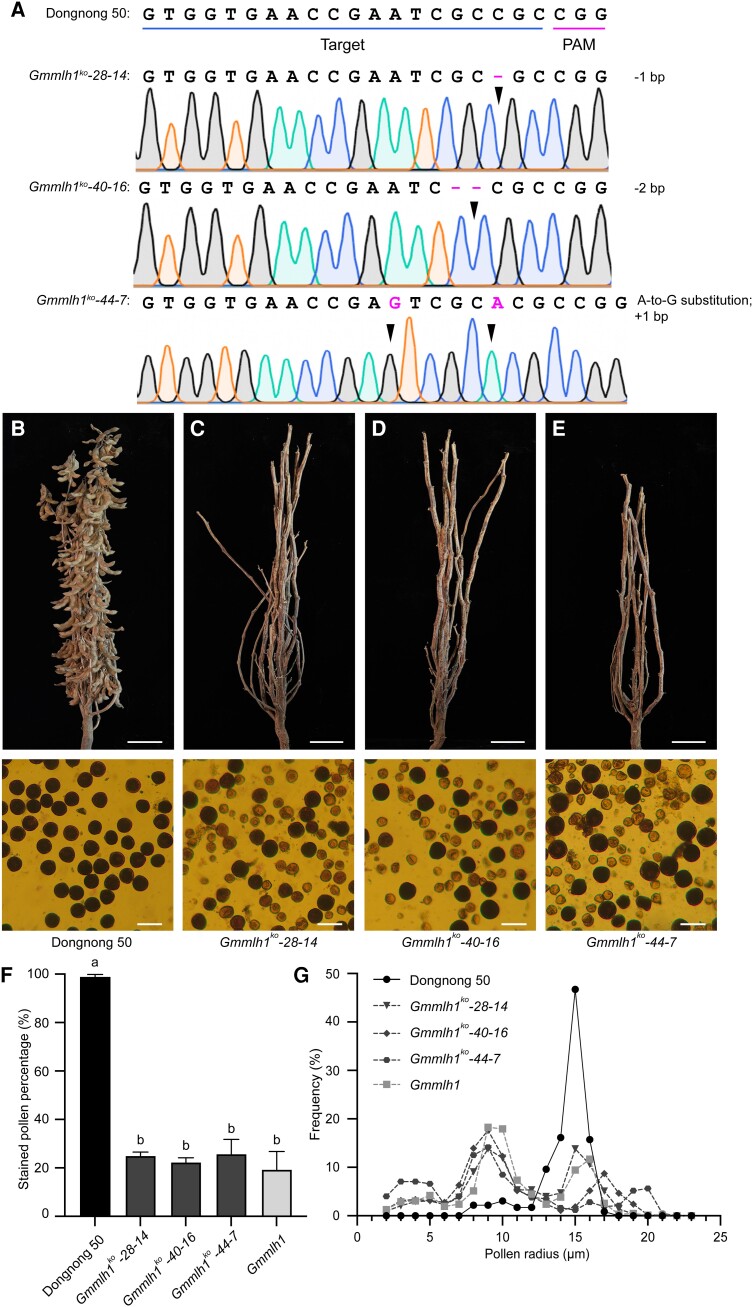
Characterization of the *GmMLH1* knockout alleles edited by CRISPR/Cas9. **A)** Sequence comparison among Dongnong 50 and the *Gmmlh1^ko^* mutant lines. The protospacer adjacent motif sequence and the target sequence are indicated by magenta and blue underlining, respectively. The mutation sites are indicated by black arrowheads. **B–E)** Morphology of mature plants (upper) and pollen grains with 1% I_2_-KI solution (lower) of Dongnong 50, *Gmmlh1^ko^-28-14*, *Gmmlh1^ko^-40-16*, and *Gmmlh1^ko^-44-7* mutants. Scale bars = 10 cm (upper), scale bars = 50 µm (lower). **F)** Percentages of the stained pollen grains in Dongnong 50 (*n* = 1,662), *Gmmlh1^ko^-28-14* (*n* = 2,238), *Gmmlh1^ko^-40-16* (*n* = 1,575), *Gmmlh1^ko^-44-7* (*n* = 2,271), and *Gmfms1* (*n* = 4,364); data shown are means ± SD of 3 replicate plants. One-way ANOVA followed by a post-hoc Tukey’s honestly significant difference (Tukey's HSD, *P* < 0.05) test was performed. The α for statistical significance was set to 0.05. The different lowercase letters indicate significant differences among means. **G)** Frequencies of pollen grain size for Dongnong 50 (*n* = 229), *Gmmlh1^ko^-28-14* (*n* = 783), *Gmmlh1^ko^-40-16* (*n* = 538), *Gmmlh1^ko^-44-7* (*n* = 424), and *Gmfms1* (*n* = 624).

To further verify that loss of *GmMLH1* was responsible for the *Gmfms1* mutant phenotype, we used CRISPR/Cas9-based genome engineering to knockout the *GmMLH1* gene in a Chinese soybean cultivar, Dongnong 50. Fifty-eight T_0_ transgenic lines were obtained, eight of which were *bar*-resistant (as detected using test strips). Three independent T_0_ transgenic lines (*Gmmlh1^ko^-28*, *Gmmlh1^ko^-40*, and *Gmmlh1^ko^-44*) were effectively edited and carried heterozygous mutations at the target sites. The mutated plants (*Gmmlh1^ko^-28-14*, *Gmmlh1^ko^-40-16*, and *Gmmlh1^ko^-44-7*) carried a 1-bp deletion at the 80th nucleotide; a 2-bp deletion at the 78th–79th nucleotides; and an A-to-G substitution at the 75th nucleotide plus a 1-bp insertion at the 81st nucleotide, respectively (Fig. 3A). All these mutations caused shifts in the coding frame, leading to the production of a truncated GmMLH1 protein. Similar to the *Gmfms1* mutant, the *Gmmlh1* knockout plants exhibited severely reduced fertility with deformed pollen grains (Fig. 3B–E, Supplementary Fig. S2). The percentages of darkly stained pollen grains in *GmMLH1* knockout mutants from T_2_ generation lines were 25.04 ± 1.51% (*Gmmlh1^ko^-28-14*), 22.48 ± 1.81% (*Gmmlh1^ko^-40-16*), and 25.80 ± 5.21% (*Gmmlh1^ko^-44-7*) (Fig. 3F). The radius of most of the pollen grains in the *Gmmlh1* knockout mutants was about 8 to 10 µm, while the radius was 14 to 16 µm in Dongnong 50 (Fig. 3G). These results demonstrated that the mutation in the *GmMLH1* gene was responsible for the severe reduction of fertility in the *Gmfms1* mutant.

### GmMLH1 is a homolog of the MutL protein in soybean

Based on the reference sequence in the plant genomic resource database (https://phytozome.jgi.doe.gov/pz/portal.html), we obtained the 2,184-bp full-length cDNA sequence of the *GmMLH1* gene, which contained 16 exons and encoded a protein 727 amino acids in length (Fig. 2B and D). Three conserved domains were identified in the putative protein: a Histidine kinase-like ATPase (HATPase) domain (residues 32 to 158), a DNA mismatch repair domain (residues 223 to 345), and a MutL C-terminal domain (residues 439 to 727) (Fig. 2D). The GmMLH1 protein was highly similar to MLH1 proteins from Arabidopsis (71.8% identity and 84.2% positive), human (*Homo sapiens*) (48% identity and 68% positive), and yeast (46% identity and 68% positive). Across all aligned species, the HATPase domain contained four conserved motifs, namely uubEuuaNouDA, uxuxDNGxGuxbaauxxuu, uGxxGxouxSxxxuoxbuTuxT, and Tx_n_GT, which are essential for ATP binding and/or hydrolysis (Supplementary Fig. S3A). The C-terminal domains of all MLH1 homologs harbored a highly conserved FERC motif, which may facilitate interaction with other mismatch repair (MMR) proteins (Reyes et al. 2015).

Phylogenetic analysis of the MutL homologs showed that the representative MutL family was divided into 3 groups: MLH1, PMS1 (postmeiotic segregation 1), and MLH3 (Supplementary Fig. S3B). Each group was clearly clustered into 2 clades (eudicots and monocots), indicating that the time of functional differentiation of the MutL homologs occurred before the differentiation of eudicots and monocots (Supplementary Fig. S3B). Synteny analysis revealed that the two syntenic blocks of *GmMLH1* flanking sequence were detected on the Chromosome 6 of *G. max*; however, these 2 blocks were separated by the unrelated synteny blocks (Supplementary Fig. S4). We then searched the full-length amino acid sequence of GmMLH1 against the plant genomics resource database using BLASTp. A 69-aa peptide encoded by *Glyma.19G059900* had 94.7% similarity to the 501 to 555 residues of the GmMLH1 C-terminus, but no synteny blocks were detected within this region. Based on the GeneAtlas v2 database (http://geneatlas.roslin.ed.ac.uk), the *Glyma.19G059900* gene is not expressed. We speculated that *Glyma.19G059900* may have lost its function. Together, these results suggested that *GmMLH1* is a single-copy soybean *MutL* homolog gene.

*GmMLH1* was ubiquitously expressed in leaves, flowers, buds, stems, and roots, but was particularly highly expressed in the buds (Supplementary Fig. S3C). Subcellular localization assays in the leaf epidermal cells of *Nicotiana benthamiana* showed that the GmMLH1-GFP signal was predominantly localized in the nucleus, overlapping with the nuclear localization marker AHL22-mCherry (Yun et al. 2012), whereas free GFP was distributed throughout the nucleus and cytoplasm (Supplementary Fig. S3D). These results suggested that the GmMLH1 protein was localized in the nucleus.

### Meiosis is disrupted in the *Gmmlh1* mutant

**Figure 4.**
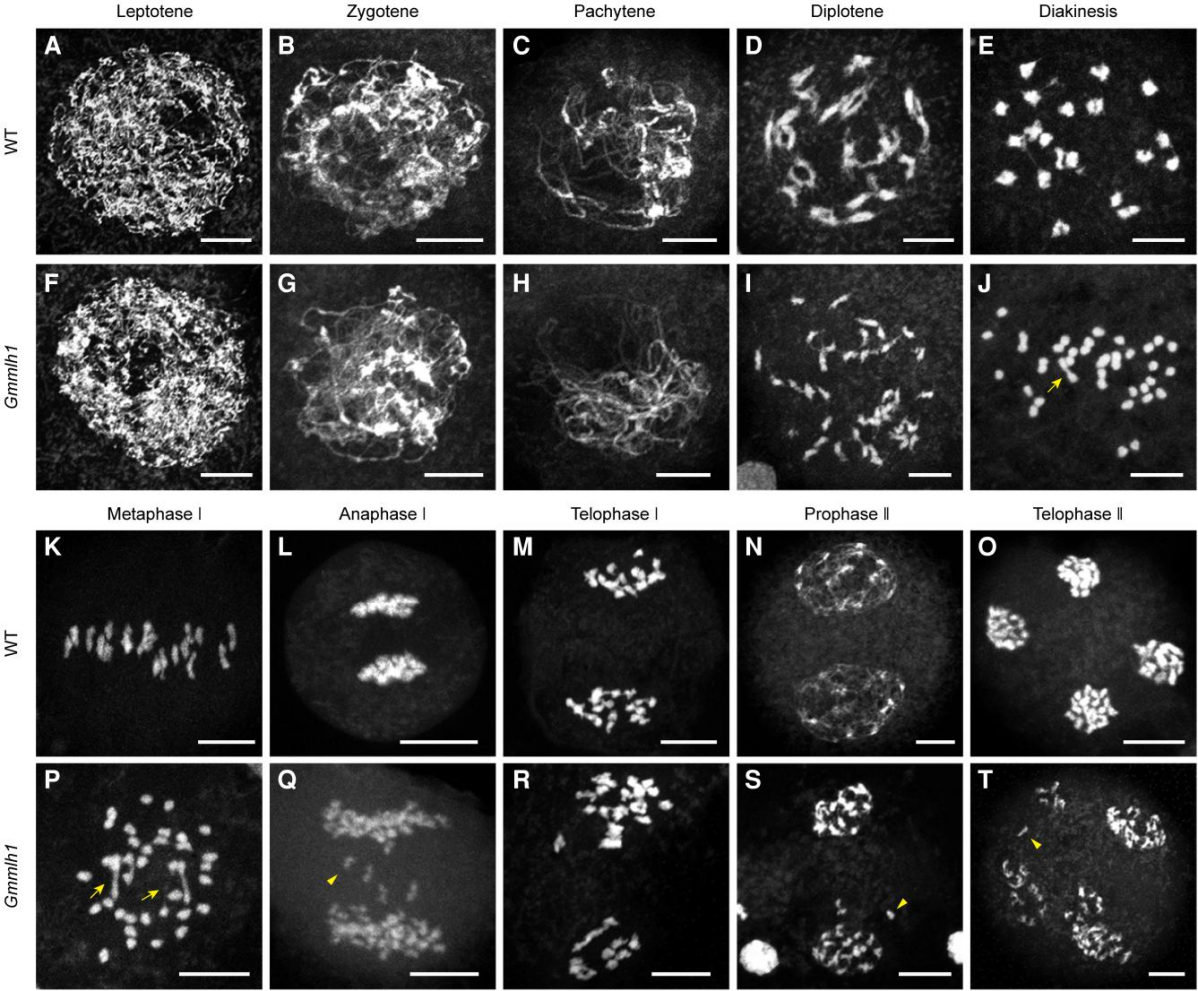
Meiotic chromosome behaviors in the pollen mother cells of the wild type and the *Gmmlh1* mutant. **A–E**, **K–O**) Wild type (WT) and **F–J**, **P–T**) *Gmmlh1* mutant. **A**, **F**) Leptotene. **B**, **G**) Zygotene. **C**, **H**) Pachytene. **D**, **I**) Diplotene. **E**, **J**) Diakinesis. **K**, **P**) Metaphase I. **L**, **Q**) Anaphase I. **M**, **R**) Telophase I. **N**, **S**) Prophase II. **O**, **T**) Telophase II. Arrows indicate bivalents, arrowheads indicate univalents. Scale bars = 10 µm. Chromosomes are stained with DAPI.

To identify the specific dysfunctions underlying the sterility of the *Gmmlh1* mutant, we investigated meiotic chromosome behaviors in the wild type and *Gmmlh1* male meiocytes. From leptotene to pachytene, the chromosome behavior of the *Gmmlh1* mutant was similar to that of the wild type (Fig. 4A–C and F–H). At diplotene, we observed that most homologous chromosomes were separated from one another in the *Gmmlh1* mutant, while all the homologous chromosomes were linked by chiasmata in the wild type (Fig. 4D and I). At diakinesis, the homologous chromosomes in wild-type meiocytes condensed to form 20 bivalents, which were organized by the spindle and aligned along the equatorial plane at Metaphase I (Fig. 4E). The homologous chromosomes migrated to opposite poles during Anaphase I and Telophase I (Fig. 4K–M). In contrast, the *Gmmlh1* meiocytes had abnormal univalents that were dispersed throughout the nucleus from diakinesis to metaphase I (Fig. 4J and P). The univalents were randomly distributed, leading to unequal chromosome segregation during Anaphase I and Telophase I in the *Gmmlh1* mutant (Fig. 4Q and R). During the 2nd meiotic division, sister chromatids separated from one another, resulting in the formation of tetrads with 4 haploid daughter cells in the wild type, Fig. 4N and O). However, the *Gmmlh1* meiocytes exhibited unequal chromosome segregation, leading to daughter cells with abnormal chromosome numbers (Fig. 4S and T). Consequently, unbalanced chromosome segregation in *Gmmlh1* mutant tetrads led to aberrations in microspore development and decreased pollen fertility in the *Gmmlh1* mutant. Similar abnormal chromosome behaviors were observed in the *Gmmlh1* knockout mutants (*Gmmlh1^ko^-28-14*, *Gmmlh1^ko^-40-16*, and *Gmmlh1^ko^-44-7*) (Supplementary Fig. S5). Thus, these results demonstrated that *GmMLH1* is required for normal meiosis progression.

### Bivalent formation defects in the *Gmmlh1* mutant

**Figure 5.**
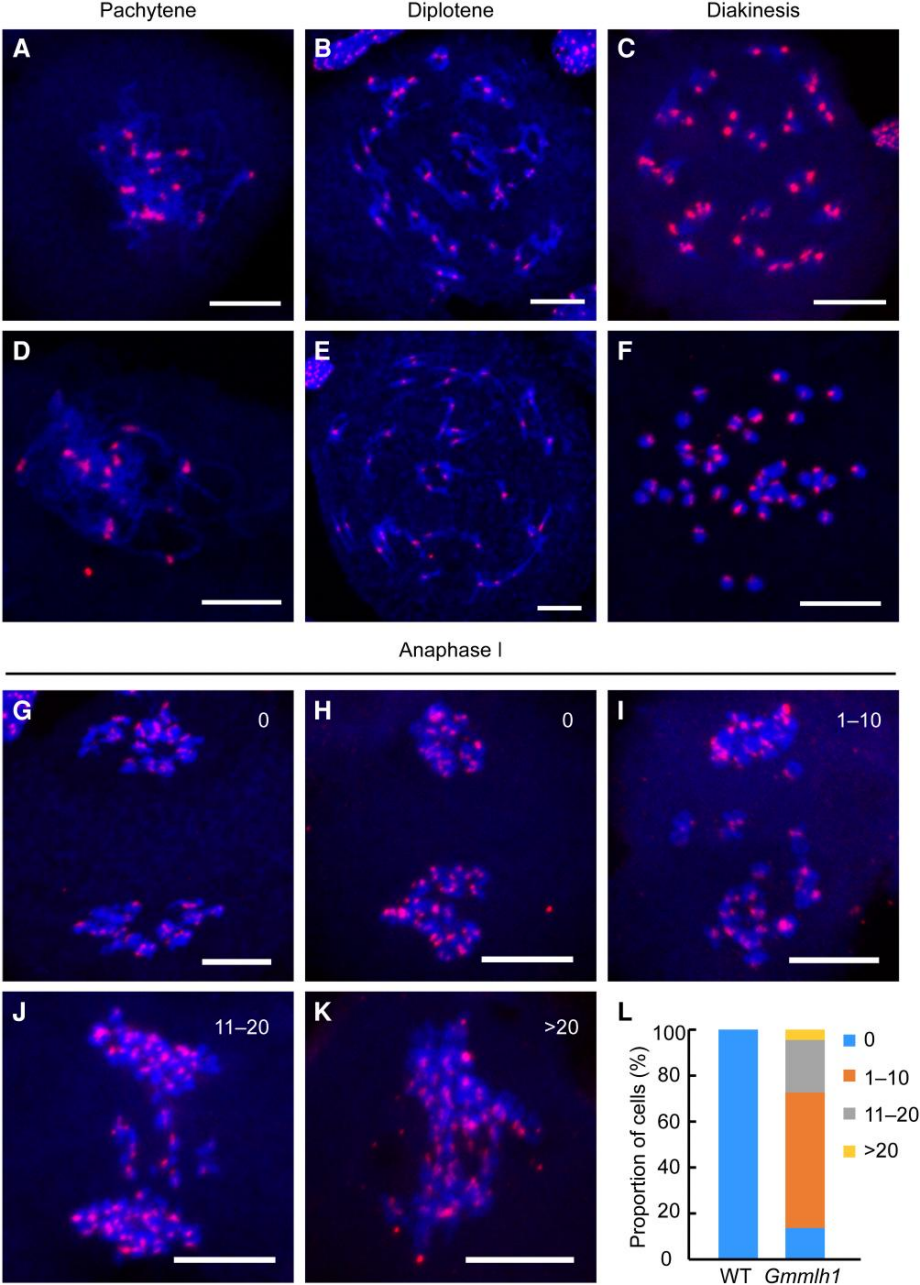
Fluorescent in situ hybridization analysis showing the formation of univalent and lagging chromosomes in the *Gmmlh1* mutant. Centromere signals in pachytene **A, D)**, diplotene **B, E)**, diakinesis **C–F)**, and Anaphase I **G–K)** cells from the wild type **A–C, G)** and the *Gmmlh1* mutant **D–F, H–K)**. The Arabic numbers indicate the number of lagging chromosome signals in Anaphase I cells **G–K)**. Scale bars = 10 µm. **L)** The percentage of cells with a different number of lagging chromosomes. Four levels of lagging chromosomes (0, 1 to 10, 11 to 20, and >20) were defined according to the number of lagging chromosomes per cell in the wild type (*n* = 13) and the *Gmmlh1* mutant (*n* = 44).

**Figure 6.**
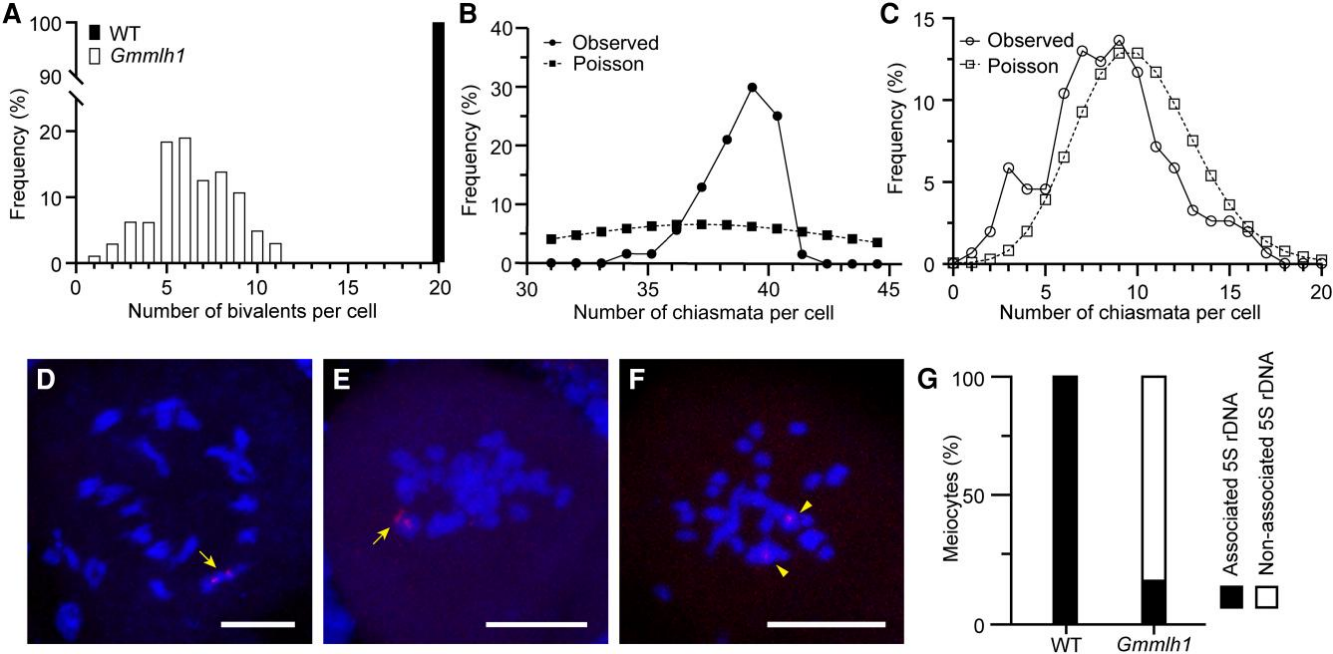
GmMLH1 participates in interference-sensitive crossover formation. **A)** Frequency of numbers of bivalents per cell in the wild type (WT; *n* = 123) and the *Gmmlh1* mutant (*n* = 158). **B, C)** Observed (dots) and predicted (squares) Poisson distributions of chiasmata in pollen mother cells of the wild type **B**; *n* = 123) and the *Gmmlh1* mutants **C**; *n* = 158). **D–F)** FISH detection of chromosome interactions using the 5S rDNA probe in diakinesis cells in the wild type **D**; *n* = 31) and the *Gmmlh1* mutant **E, F**; *n* = 52). **G)** The percentage of associated or nonassociated 5S rDNA signals on the chromosomes in Anaphase I cells in both the wild type and *Gmmlh1* mutant. Arrows indicate bivalents, and arrowheads indicate univalents. Scale bars = 10 µm.

To further explore the mechanisms underlying the meiosis defects in the *Gmmlh1* mutant, we used the Cy3-labeled centromere probe CentGm to reveal the detailed morphology of chromosome associations from pachytene to diakinesis in both the wild type and *Gmmlh1* mutant. We did not observe a difference between the wild type and *Gmmlh1* mutant at pachytene (Fig. 5A and D). At diplotene, the CentGm signals located on each homologous chromosome were clearly paired in the wild type; however, some CentGm signals were unpaired in the nucleus of the *Gmmlh1* mutant (Fig. 5B and E). At diakinesis, 20 paired CentGm signals were located in each homologous chromosome in the wild type, while few paired CentGm signals were detected in the *Gmmlh1* mutant (Fig. 5C and F). Comparative analysis showed that there were 68.00% fewer bivalents per cell in the *Gmmlh1* mutant (an average of 6.40 ± 2.23 per meiocyte) than in the wild type (an average of 20 ± 0.00 per meiocyte; *P* < 0.0001, Student's unpaired *t*-test) (Fig. 6A), indicating a potential loss of COs. These results demonstrated that the bivalent formation was affected in the *Gmmlh1* mutant, leading to the reduction of COs. In addition, compared with the wild type, the *Gmmlh1* mutant exhibited increased lagging chromosomes at Anaphase Ⅰ (Fig. 5G–K). Only 13.64% of *Gmmlh1* mutant cells showed no lagging chromosomes, and 86.36% of mutant cells exhibited different numbers of lagging chromosomes at Anaphase Ⅰ (Fig. 5L).

### *GmMLH1* is involved in interference-sensitive CO formation

To explore how *GmMLH1* affects CO formation, we quantified chiasma frequency and distribution according to chromosome morphology (Moran et al. 2001, Huang et al. 2022). The mean chiasmata number in the *Gmmlh1* mutant (8.41 ± 3.28 per meiocyte) decreased 78.16% relative to the wild type (38.50 ± 1.41 per meiocyte) (Fig. 6B and C). The chiasmata distribution per cell in the wild type deviated significantly from a Poisson distribution (*P* = 0.0061 < 0.01, Kolmogorov–Smirnov test), whereas the distributions of residual chiasmata on chromosomes in the *Gmmlh1* mutant were consistent with the predicted Poisson distribution. These results suggested that residual chiasmata found in *Gmmlh1* were randomly distributed among cells (*P* = 0.8407 > 0.01, Kolmogorov–Smirnov test) and correspond to interference-insensitive COs (Fig. 6B and C). These findings provided strong evidence that the *GmMLH1* plays an important conserved role in the formation of interference-sensitive COs.

The 5S rDNA sequence is a tandem repeat present only on the short arm of Chromosome 19 in soybean (Gottlob-McHugh et al. 1990). During diakinesis, 2 associated 5S rDNA signals were clearly observed on the bivalent in all wild-type meiocytes (Fig. 6D and G). However, only 15.38% of the meiocytes showed 2 associated signals on the bivalent, while the remaining meiocytes exhibited 2 separated 5S rDNA signals on univalents in the *Gmmlh1* mutant (Fig. 6E–G). Based on the morphology of chromosome, we further examined the chiasmata on Chromosome 19, and found that the COs in the *Gmmlh1* mutant were reduced to 13.89%.

### GmMLH1 interacts with GmMLH3 in soybean

**Figure 7.**
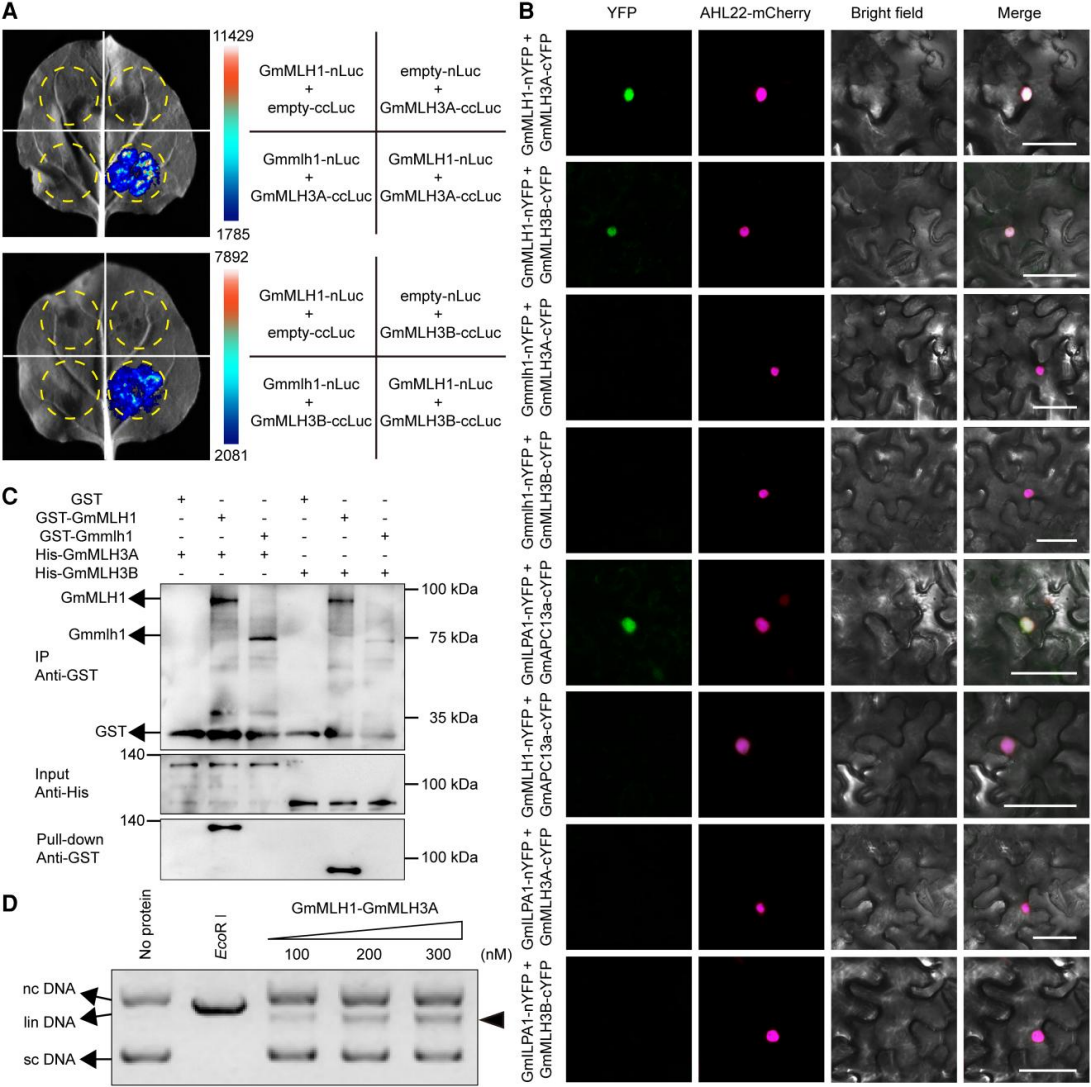
GmMLH1 interacts with GmMLH3 and shows endonuclease activity. **A)** Split-LUC assays showing interactions between GmMLH1 and GmMLH3. GmMLH1 was fused to an N-terminal fragment of luciferase (nLuc), while GmMLH3A/B were fused to a C-terminal fragment of luciferase (ccLuc). The dashed circles indicate the areas infiltrated with *A. tumefaciens* harboring the constructs corresponding to those listed in the right panel. The luminescence intensity is quantified and presented as a heatmap. **B)** BiFC assays demonstrating the physical interactions between GmMLH1 and GmMLH3. The combination of GmILPA1-nYFP + GmAPC13a-cYFP served as the positive control, AHL22-mCherry was used as the nuclear marker. YFP: Yellow fluorescent protein. Scale bars = 50 µm. **C)** Interaction between GmMLH1 and GmMLH3 detected using the pull-down assays. GST-GmMLH1 with His-GmMLH3A or His-GmMLH3B were pulled-down using GST resin and examined with anti-GST and anti-His. The “+” symbol indicates the addition of the protein to the reaction, while the “−” symbol indicates the substance was not added. IP, immunoprecipitation. **D)** GmMLH1–GmMLH3A displays the endonuclease activity on pUC19. In Lanes 3, 4, and 5, GmMLH1–GmMLH3A is at 100, 200, and 300 nM, respectively. The supercoiled DNA (sc DNA), linear DNA (lin DNA), and nicked circles DNA (nc DNA) are indicated with arrows. The product digested by GmMLH1–GmMLH3A is shown with arrowhead.

To further investigate the function of GmMLH1 in soybean meiosis, we explored the possible interaction between GmMLH1 and GmMLH3 in soybean. The soybean genome contains a homolog of *MLH3* (*Glyma.01G158400.1*, *GmMLH3A*) and a MLH3-related gene (*Glyma.11G086300.1*, *GmMLH3B*), which share the highest similarity with *AtMLH3* (*AT4G35520.1*) (Supplementary Fig. S6A). Similar to GmMLH1, both GmMLH3A and GmMLH3B have 3 identical conserved domains (Supplementary Fig. S6B and D). In addition, the conserved metal binding motif DQHA(X)_2_E(X)_4_E was contained within the C-terminal domain of GmMLH3 (Supplementary Fig. S6D). The *GmMLH3A* and *GmMLH3B* were constitutively expressed in various soybean tissues, and the expression patterns of *GmMLH3A* and *GmMLH3B* were similar to the expression patterns of *GmMLH1* (Supplementary Fig. S6C). Split-luciferase complementation (Split-LUC) assays showed that GmMLH1 interacts with both GmMLH3A and GmMLH3B when transiently expressed in *N. benthamiana* leaves (Fig. 7A). These interactions have been further verified using bimolecular fluorescence complementation (BiFC) assays in *N. benthamiana*. The GmMLH1-nYFP and GmMLH3A/B-cYFP proteins appeared close together and produced interaction signals in the nuclei of the *N. benthamiana* cells, just like the signals shown in the positive control (Fig. 7B). Furthermore, we conﬁrmed the physical interaction of GmMLH1 and GmMLH3A/B using an in vitro pull-down assay (Fig. 7C). These results demonstrated that GmMLH1 and GmMLH3 physically interact in vivo and in vitro.

To test whether the GmMLH1-GmMLH3 complex displayed an endonuclease activity, we performed a nuclease assay on pUC19 with purified GmMLH1–GmMLH3A. The pUC19 incubated with GmMLH1-GmMLH3A showed concentration-dependent conversion to the nicked form (Fig. 7D). Taken together, our results indicated that GmMLH1 and GmMLH3 might function as a heterodimer, which contains intrinsic endonuclease activity.

### GmMLH1 functions in somatic DNA damage repair

**Figure 8.**
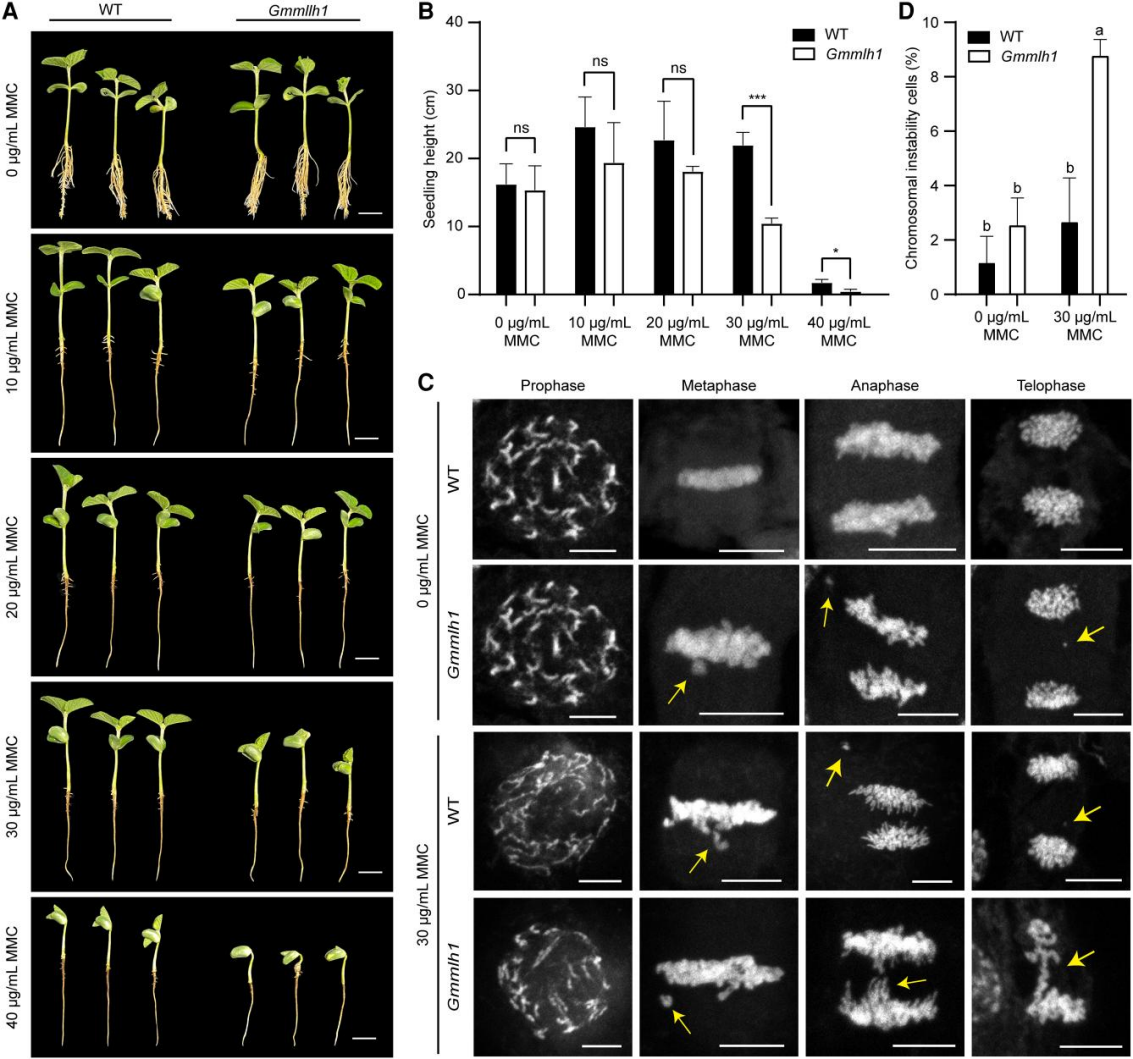
GmMLH1 is required for somatic DNA repair. **A)** Comparison of the WT and the *Gmmlh1* mutant seedlings with MMC treatment. Images were digitally extracted for comparison. Scale bars = 10 cm. **B)** Statistical analysis of the height of wild-type and *Gmmlh1* seedlings without and with MMC treatments. Asterisks indicate statistically significant differences (***P < 0.001, *P < 0.05, Student's unpaired *t*-test); ns, not significant. All data shown are means ± SDs. **C)** Mitotic chromosome behaviors observed in the root tips of the wild type and *Gmmlh1* mutant at prophase, metaphase, anaphase, and telophase. Arrows indicate chromosome fragments and bridges. Scale bars = 10 µm. **D)** Statistical analysis of the cells with chromosomal instability in root apical meristem of the wild type and *Gmmlh1* mutant without and with MMC treatments. One-way ANOVA followed by a post-hoc Tukey’s honestly significant difference (Tukey's HSD) test was performed. The α for statistical significance was set to 0.05. The different lowercase letters indicate significant differences among means. All data shown are means ± SDs.

To determine the involvement of *GmMLH1* in somatic DNA damage repair, we assessed the sensitivity of *Gmmlh1* to DSB-inducer reagent, Mitomycin C (MMC). The 3-day-old seedlings from both wild-type plants and the *Gmmlh1* mutant were treated with different concentrations of MMC (0, 10, 20, 30, and 40 µg/mL). Both wild type and *Gmmlh1* mutant exhibited growth retardation in their secondary roots when grown in culture medium supplemented with MMC (Fig. 8A). However, no significant differences in height were observed between the wild-type seedlings (*n* = 9, 9, 9) and *Gmmlh1* mutants (*n* = 13, 9, and 11) grown in Hoagland culture supplemented with 0 to 20 µg/mL MMC (*P* > 0.05, Student's unpaired *t*-test) (Fig. 8B). The height of the *Gmmlh1* mutant seedling (*n* = 10 and 7) was significantly lower than the wild type (*n* = 9 and 9) when grown in Hoagland culture containing 30 µg/mL (*P* < 0.001, Student's unpaired *t*-test) and 40 µg/mL (*P* <0.05, Student's unpaired *t*-test) of MMC (Fig. 8B).

We further examined mitotic progression in both wild-type and *Gmmlh1* seedlings untreated and treated with 30 µg/mL MMC, assessing the frequency of chromosomal instability during telophase (Fig. 8C and D). In wild-type seedlings, the frequency of abnormal mitotic cells with chromosome fragments and bridges increased by 2.24-fold in treated seedlings (2.67 ± 0.62%, *n* = 274) compared to untreated seedlings (1.19 ± 0.99%, *n* = 300; *P* = 0.1932 > 0.05, ANOVA). However, in *Gmmlh1* mutants, the frequency significantly increased by 3.45-fold in treated seedlings (8.79 ± 0.62%, *n* = 274) compared to untreated counterparts (2.25 ± 1.04%, *n* = 265; *P* < 0.0001, ANOVA) (Fig. 8D). These results suggested that *Gmmlh1* mutants are more sensitive to MMC treatment, suggesting *GmMLH1* is involved in somatic mismatch repair function.

## Discussion

### Conserved function of the *GmMLH1* gene in soybean

MLH1 homologs have been identified in various species, including yeast, ﬂies (*Drosophila melanogaster*), mice (*Mus musculus*), humans, Arabidopsis, tomato (*Solanum lycopersicum*), and rice (Prolla et al. 1994; Baker et al. 1996; Hunter and Borts 1997; Ellison et al. 2004; Dion et al. 2007; Lhuissier et al. 2007; Vimal et al. 2018; Mao et al. 2021). These studies have shown that MLH1 has a conserved function in both MMR and meiotic CO formation. In Arabidopsis and rice, it has been shown that the mutation of the *MLH1* gene leads to abnormal chromosome behaviors in pollen mother cells, including an increase in univalents and a decrease in COs (Dion et al. 2007; Xin et al. 2021). Additionally, studies have revealed that OsMLH1 interacts with OSMLH3 to form a heterodimer, which is essential for the formation of interference-sensitive COs during meiosis (Mao et al. 2021; Xin et al. 2021). Our work also demonstrated the physical interaction between GmMLH1 and GmMLH3 in soybean using in vivo and in vitro assays. Furthermore, we found that the *Gmmlh1* mutant was more sensitive to MMC treatment compared to the wild type, which indicates that *GmMLH1* is involved in somatic DNA damage repair. In summary, our findings reveal a function of *MLH1* in meiosis and MMR, and will provide a useful information for further research to broaden our understanding of the functions of *MLH1* in plants.

### Severe reduction in fertility of the *Gmmlh1* mutant

Seed-setting rates of approximately 20%, 13.7%, and 14% have been reported for the *Atmlh1*, *indica Osmlh1*, and *japonica Osmlh1* mutants, respectively (Dion et al. 2007; Mao et al. 2021; Xin et al. 2021). In this study, we observed a more severe reduction in fertility in the soybean *mlh1* mutant compared to the *mlh1* mutants of other plant species. The severity of sterility might be related to the number of bivalents in meiotic mutants due to the random segregation of univalents (Couteau et al. 1999). For example, the random chance of equilibrated chromosome segregation in Arabidopsis with 10 univalents is about 3.13% (*P*_(*n*__=__5)_ = [1/2]^5^), as observed in the *Atdmc1* (*A. thaliana disrupted meiotic cDNA 1*) mutant (Couteau et al. 1999). In maize, the 20 univalents of the *spo11-1* mutant lead to the expected chance of 0.10% (*P*_(*n*__=__10)_ = [1/2]^10^) residual fertility (Ku et al. 2020). These results indicate that the low chromosome number of Arabidopsis allows the production of few euploid spores and gametes, despite an achiasmatic meiosis, by the random segregation of univalents. In the *Gmmlh1* mutant, although an average of 6.40 ± 2.23 bivalents per meiocyte were observed, the expected chance of having the correct set of 20 chromosomes was still extremely low (*P*_(*n*__=__20–6.4)_ = [1/2]^13.6^ ≈ 0.008%), which was obviously lower than that in Arabidopsis and rice. Furthermore, the genome size may also affect the fertility of *mlh1* mutants. The soybean *Gmmlh1* mutant, possessing a larger genome size of 1.1 to 1.15 Gb compared to Arabidopsis (125 Mb) and rice (430 Mb), may accumulate more endogenous DNA damage, potentially leading to a greater reduction in fertility than that observed in its Arabidopsis and rice counterparts. Our study also found that the embryo sacs showed a more severe reduction in fertility than that in pollen grains. The possible difference in sterility between male and female might be associated with heterochiasmy (Saini et al. 2020).

### Substantial Class I CO reduction in the *Gmmlh1* mutant

The residual chiasmata in the *Gmmlh1* mutant were randomly distributed, similar to those in other Class I CO mutants such as *Atmlh3*, *Oshei10* (*Oryza sativa human enhancer of invasion 10*), and *Osmsh4*/*5* (Jackson et al. 2006; Wang et al. 2012a; Luo et al. 2013; Wang et al. 2016). This similarity suggests that GmMLH1 is involved in the formation of Class I meiotic COs in soybean. We still observed approximately 14% residual COs, corresponding to an average of 6.40 ± 2.23 bivalents per meiocyte in the *Gmmlh1* mutant. The residual COs may originate from the Class Ⅱ CO pathway, consistent with a previous report on the *japonica Osmlh1* mutant (Xin et al. 2021). However, we observed a reduction of more than 75% in the COs in the *Gmmlh1* mutant. This reduction is greater than those previously reported, including a 50% reduction in yeast (Argueso et al. 2004), a reduction of approximately 60% in Arabidopsis (Jackson et al. 2006), and a 47% reduction in rice (Xin et al. 2021). These findings suggest that species-specific, MLH1-dependent CO formation mechanisms may lead to variations in CO frequency among different species. Notably, the soybean genome has approximately 57% heterochromatic regions, which are rich in repetitive sequences and suppressed for meiotic recombination (Schmutz et al. 2010; Henderson 2012). Therefore, the differences in Class I meiotic COs may be associated with heterochromatic size among these species. More detailed research is needed to clarify the differences in CO distribution and frequency among different species.

Low recombination frequencies often resulted in higher percentage of univalent formation, leading to higher chances of unequal chromosome segregation and aneuploidy in spores. Consequently, progenies derived from aneuploid spores might produce aneuploid plants. Recently, Pochon et al. (2023) have observed that aneuploid Arabidopsis plants in progenies derived from crosses involving the *asy1* (*meiotic asynaptic mutant 1*), which exhibited univalents in male meiocytes. This finding paved the way for studying aneuploid soybean using *Gmmlh1* mutant. Further investigation for *MLH1* function is required to elucidate the molecular mechanisms underlying meiotic recombination in plants.

## Materials and methods

### Plant materials and growth conditions

The *Gmmlh1* mutant was obtained by treating the Williams 82 soybean (*G. max*) genotype with a chemical mutagen (ethyl methanesulfonate) as described before (Wang et al. 2020). All plant materials used for cytological analysis were grown in the experimental fields of the Northeast Institute of Geography and Agroecology (Changchun, China); plants used for reverse transcription quantitative PCR (RT-qPCR) analysis were grown in a growth chamber at 28 °C with 50% humidity and a photoperiod of 14/10-h light/darkness. The *N. benthamiana* plants were grown in a growth chamber at 23 °C with 80% humidity and a photoperiod of 16/8-h light/darkness.

### Bulked segregant analysis based on genomic DNA resequencing

The F_2_ plants derived by crossing the heterozygous *GmMLH1*/*Gmmlh1* plants and Hedou 12 soybean cultivar were used to map the *GmMLH1* gene. Bulked segregant analysis was performed as previously described (Feng et al. 2019; Tang et al. 2020), the common control pool sequence data previously published by our laboratory was used as wild-type pool for BSA (Tang et al. 2020; Zhou et al. 2021). Libraries were constructed using mutant DNA samples, and whole-genome resequencing was performed using the Illumina HiSeq 2500 platform (Illumina, USA). Roughly 30× genome sequences sample were generated. SNPs and small InDels were identified by aligning the sequence reads of bulked DNA samples to the *Glycine max Wm82.a2.v1* reference genome using the Burrows–Wheeler Aligner (v0.7.16a) software (Li and Durbin 2009; Schmutz et al. 2010). An average SNP index for the sterile bulked samples was calculated using a 200-kb sliding window with a step size of 50 kb. Genomic regions with SNP index >0.9 were identified as candidate regions.

### Genotype analysis by CAPS

The specific oligos for CAPS markers were designed according to the SNPs between the wild-type and mutant sequences of *GmMLH1* using the web-based tool dCAPS Finder 2.0 (http://helix.wustl.edu/dcaps/dcaps.html) (Neff et al. 2002). The PCR product was digested using *Mse* Ⅰ (New England Biolabs, USA) in 37 °C for 1 h. The digested samples were analyzed using 2.0% (w/v) agarose gel in TAE buffer. Primers used in this assay are listed in Supplementary Table S3.

### Preparation of embryo sacs for WCLSM

Embryo sacs were prepared for WCLSM as previously described (Lu et al. 2020). Buds collected at various developmental stages were fixed in Carnoy's fixative (75% ethanol and 25% acetic acid) overnight at room temperature. The fixed ovaries were dissected, and transferred to 70% ethanol. The samples were then hydrated sequentially using 50%, 30%, and 15% ethanol, and distilled water; stained with 1% eosin Y for about 8 h; and washed several times in distilled water until colorless. The samples were next treated with citric acid disodium hydrogen phosphate buffer (0.1 mol/L, pH = 5.0) for 8 h, followed by Hoechst staining in darkness at 25 °C for 24 h. After staining, samples were washed 3 times with distilled water, and then dehydrated using an ethanol series (15%, 30%, 50%, 70%, 85%, 95%, and 100%). The dehydrated samples were incubated in a 1:1 solution of ethanol and methyl salicylate at room temperature for 1 h, cleared 3 times in methyl salicylate (2, 2, and 15 h), and preserved in methyl salicylate. The embryo sacs were captured using a Nikon C2 laser scanning confocal microscope (Nikon, Japan).

### Vector construction and plant transformation

To clone the full-length coding sequence of *GmMLH1*, total RNA was initially isolated from young buds of Williams 82 using Trizol reagent (Tiangen, China). First-strand cDNA was then synthesized from 1 µg of total RNA using the RT-PCR kit (TransGene, China). A 3-step PCR was conducted to amplify specific DNA fragments (TOYOBO, Japan). The PCR products were ligated into the pGEM-T EASY vector (Promega, USA), and the recombinant vector was transformed into *Escherichia coli* DH5α competent cells (Transgene, China). Finally, the plasmid DNA was purified and sequenced.

To generate the *GmMLH1* knockout line, single-guide RNA (sgRNA) targeting *GmMLH1* were designed (GmMLH1-CR-F and GmMLH1-CR-R) and were artificially synthesized (Sangon, China). The sgRNA were cloned into the pVK005-04-GmU6-2-GmUbi3 vector following *Bsp*Q Ⅰ (New England Biolabs, USA) digestion (Du et al. 2016). The knockout construct was introduced into *Agrobacterium tumefaciens* strain EHA105, which was then transformed into cotyledonary explants of soybean cultivar Dongnong 50 with *A. tumefaciens*-mediated transformation (Paz et al. 2006; Wang et al. 2020).

For subcellular localization analysis, the full-length cDNA without stop codon of *GmMLH1* was amplified from Williams 82 using GmMLH1-sl-F and GmMLH1-sl-R primer pair. The PCR amplicons were cloned into the pCAMBIA1300-*eGFP* vector between the restriction endonuclease sites *Kpn* Ⅰ and *Sal* Ⅰ (New England Biolabs, USA) to generate 35S:*GmMLH1*-*eGFP* cassettes. For bimolecular fluorescent complementation (BiFC) assays, the full-length cDNA sequences of *GmMLH1* and *GmMLH3* without stop codons were amplified. The PCR products were cloned into the *Bam*H Ⅰ (New England Biolabs, USA) site of the modified vectors pEarleygate201-nYFP and pEarleygate202-cYFP plasmids, respectively (Earley et al. 2006). For Split-LUC assays, the coding sequence of *GmMLH1* without stop codon was cloned into the *Kpn* Ⅰ and *Sal* Ⅰ (New England Biolabs, USA) sites of the pCAMBIA1300-nLuc vector, while the coding sequences of *GmMLH3* without stop codons were cloned into the *Kpn* Ⅰ and *Sal* Ⅰ (New England Biolabs, USA) sites of separate pCAMBIA1300-ccLuc vectors (Zhou et al. 2018).

For subcellular localization and BiFC assays, the resulting constructs were introduced into *A. tumefaciens* strain EHA105, and then injected into young *N. benthamiana* leaves (Wang et al. 2020). Leaves were examined using a confocal microscope (Nikon C2, Japan) after 36 to 72 h incubation. For Split-LUC assays, the resulting constructs were introduced into *A. tumefaciens* strain GV3101, and then injected into young *N. benthamiana* leaves (Wang et al. 2020). To measure luciferase activity, 1 mM luciferin was sprayed into the leaves. After incubation in the dark for 5 min, images were captured using a cooled charge-coupled device imaging apparatus (Tanon, China). All primers used in these assays are listed in Supplementary Table S3.

### Multiple sequence alignments and phylogenetic analysis

We searched the full-length amino acid sequence of GmMLH1 against the plant genomics resource database using BLASTp. All the parameters were set as default except the *E-value* (>1), and low complexity regions were filtered out. Homologous protein sequences were aligned using MEGA7 software. A phylogenetic tree was constructed with the neighbor-joining algorithm using MEGA7. The bootstrap consensus tree was inferred from 1,000 replicates (Kumar et al. 2016). Synteny analysis was performed using the webtool MCScanX (Wang et al. 2012b).

### Reverse transcription quantitative PCR (RT-qPCR) analysis

Total RNA was extracted from the roots, stems, leaves, flowers, and meiosis-stage buds of chamber-grown Williams 82 plants using Trizol reagent (Tiangen, China). All RT-qPCR analyses were performed with a SYBR Premix Ex Taq Kit (Takara, Japan) on a Stratagene MX3005P Real-Time PCR System (Agilent Technologies, USA). The qPCR cycling conditions were 40 cycles of 95 °C for 15 s, 58 °C for 30 s, and 72 °C for 30 s. All RT-qPCR analyses were performed in triplicate. The relative expression of each target gene was calculated using 2^−ΔΔCt^ method after normalization to *Actin*. Primer pairs used in this analysis are listed in Supplementary Table S2.

### Meiotic chromosome preparation and fluorescent in situ hybridization (FISH) analysis

Meiotic chromosomes were detected using 4′,6-diamidino-2-phenylindole (DAPI) as previously described (Wang et al. 2014). Young buds around 1.0 to 2.0 mm in length were collected and fixed in Carnoy's fixative for 24 h at room temperature. The fixed buds were washed with citric acid buffer 3 times and digested in an enzyme solution (2% cellulose and 3% pectinase) for 40 to 60 min. Anthers were dissected and crushed in 60% acetic acid. After flash-freezing slides in liquid nitrogen for 1 min, coverslips were removed rapidly. Prechilled Carnoy's fixative (∼50 µL) was added after another drop of 60% acetic acid spreading for 1 min. Then the slides were allowed to air-dry. The air-dried slides were stained with 7 µL DAPI solution (10 µg/mL) or used for FISH.

FISH analysis was performed as previously described with modifications (Li and Cheng 2016). In brief, the soybean 5S rDNA probe was labeled with digoxigenin using a PCR DIG probe synthesis kit (Roche, USA) and detected using antidigoxigenin-rhodamine (Roche, USA) (Gottlob-McHugh et al. 1990). The centromere probe was designed following a previous study: the 27-bp probe, comprised of oligonucleotides shared between CentGm-1 and CentGm-2, was synthesized and labeled with Cy3 at the 5′ terminal (Findley et al. 2010). Probe signals were observed, and images were captured using a Nikon C2 laser scanning confocal microscope (Nikon C2, Japan). The probe sequences are listed in Supplementary Table S2.

### Confocal microscopy imaging and fluorescence detection

Images were performed on a Nikon C2 laser scanning confocal system head on a Nikon Eclipse-Ti inverted microscope. Images were captured with Plan Apo VC 20×/0.75 lens and Plan Apo λ 100×/1.45 oil immersion objective lens (Nikon, Japan). The NIS-Element AR 4.60 software (Nikon, Japan) was employed to control image acquisition. For visualization, DAPI was excited with a 405-nm laser and its emission was detected through a 417- to 477-nm bandpass filter. The enhanced green fluorescent protein (eGFP), yellow fluorescent protein (YFP), and eosin Y fluorescence were excited with a 488-nm laser and detected using a 500- to 550-nm bandpass filter. mCherry, Cyanine3, and Rhodamine were excited with a 561-nm laser, with emissions captured through 570- to 1,000-nm bandpass filter. Z-stacks images were compiled from optical sections ranging from 0.10 to 0.85 µm. The laser power and HV settings were adjusted 5 to 10 and 60 to 90, respectively.

### Pull-down assay

Protein expression, extraction, purification, and immunoblot analysis were conducted as described by Gao et al. (2017). The sequences encoding for GmMLH1 and GmMLH1^E416*^ protein without stop codons were fused into the pGEX-4T-3-GST vector, while the sequences encoding for GmMLH3 without stop codons were fused into the pET28a-His vector. For pull-down assays, 2 µg of GST- or His-tagged proteins were mixed in 400 µL binding buffer (Sangon Biotech, China) on a rotating wheel at 4 °C for 4 h, and then incubated with GST resin at 4 °C for 2 h. For western blotting analysis, anti-GST or anti-His in 1:2,000 dilution (Abmart, China) was used to detect the eluted proteins. Primers used in this assay are listed in Supplementary Table S3.

### Endonuclease assay

The pUC19 (500 ng) was incubated in 25 µL reactions containing purified GmMLH1-GmMLH3 in 20 mM Tris-acetate (pH = 7.5), 1 mM DTT, 0.1 mg/mL BSA, 10 mM magnesium acetate, 50 mM potassium acetate, and 0.5 mM ATP for 1 h at 37 °C. The reaction products were separated by electrophoresis in 1.5% agarose gel with 0.1 µg/mL ethidium bromide run in 1× TAE buffer for 30 min at 120 V.

### MMC treatment

Both the wild-type and *Gmmlh1* seeds were germinated on a vermiculite bench in a growth room (14/10-h light/darkness, 25 °C). For the MMC treatment, the 3-day-old seedlings were transferred into Hoagland solutions containing various concentrations of MMC (0, 10, 20, 30, and 40 µg/mL). After 7 days, appropriate concentration of MMC was determined by observing the seedlings’ growth state. For mitotic cell analysis, 6-day-old seedlings were transferred into Hoagland solutions containing 30 µg/mL MMC. After 24 h, the secondary root tips of the seedlings were then collected and used for mitotic chromosomes analysis.

### Accession numbers

Accession numbers of genes in this study are listed as follows: *GmMLH1* (*Glyma.04G254900*), *GmMLH3A* (*Glyma.01G158400*), and *GmMLH3A* (*Glyma.11G086300*). Sequencing data of the *Gmmlh1* mutant pool in this article can be found in the BIG Data Center (https://bigd.big.ac.cn/gsa/index.jsp) data library under the following accession number CRA006301.

## Supplementary Material

kiae165_Supplementary_Data

## Data Availability

The data underlying this article are available in the article and in its online supplementary material.

## Supplementary data

The following materials are available in the online version of this article.

**Supplementary Figure S1.** Phenotypic assessment of the *Gmfms1* mutant at the full maturity stage.

**Supplementary Figure S2.** Statistical analysis of the phenotype of the *Gmmlh1* knockout mutants.

**Supplementary Figure S3.** Characterization of GmMLH1 protein.

**Supplementary Figure S4.** Synteny plot of chromosomes 4, 6, 13, and 14 in soybean.

**Supplementary Figure S5.** Meiotic chromosome behaviors in the pollen mother cells from the *Gmmlh1* knockout mutants.

**Supplementary Figure S6.** Characterization of GmMLH3 in soybean.

**Supplementary Table S1.** The reciprocal cross tests of Williams 82 and *Gmfms1* mutant.

**Supplementary Table S2.** CentGm and 5S rDNA probe sequences in soybean.

**Supplementary Table S3.** Primers used in this study.

## Dive Curated Terms

The following phenotypic, genotypic, and functional terms are of significance to the work described in this paper:

MLH1 Gramene: AT4G09140
MLH1 Araport: AT4G09140

MLH3 Gramene: AT4G35520
MLH3 Araport: AT4G35520

MUS81 Gramene: AT4G30870
MUS81 Araport: AT4G30870

PMS1 Gramene: AT4G02460
PMS1 Araport: AT4G02460

ASY1 Gramene: AT1G67370
ASY1 Araport: AT1G67370

GmMLH1 Gramene: Glyma.04G254900
GmMLH1 Araport: Glyma.04G254900

GmMLH3A Gramene: Glyma.01G158400
GmMLH3A Araport: Glyma.01G158400

GmMLH3B Gramene: Glyma.11G086300
GmMLH3B Araport: Glyma.11G086300

## References

[kiae165-B1] Argueso JL , Wanat J, Gemici Z, Alani E. Competing crossover pathways act during meiosis in *Saccharomyces cerevisiae*. Genetics. 2004:168(4):1805–1816. 10.1534/genetics.104.032912 15611158 PMC1448724

[kiae165-B2] Baker SM , Plug AW, Prolla TA, Bronner CE, Harris AC, Yao X, Christie DM, Monell C, Arnheim N, Bradley A, et al Involvement of mouse *Mlh1* in DNA mismatch repair and meiotic crossing over. Nat Genet. 1996:13(3):336–342. 10.1038/ng0796-336 8673133

[kiae165-B3] Baumbach J , Pudake RN, Johnson C, Kleinhans K, Ollhoff A, Palmer RG, Bhattacharyya MK, Sandhu D. Transposon tagging of a male-sterility, female-sterility gene, *St8*, revealed that the meiotic *MER3* DNA helicase activity is essential for fertility in soybean. PLoS One. 2016:11(3):e0150482. 10.1371/journal.pone.0150482 26930200 PMC4773125

[kiae165-B4] Börner GV , Kleckner N, Hunter N. Crossover/noncrossover differentiation, synaptonemal complex formation, and regulatory surveillance at the leptotene/zygotene transition of meiosis. Cell. 2004:117(1):29–45. 10.1016/S0092-8674(04)00292-2 15066280

[kiae165-B5] Cannavo E , Sanchez A, Anand R, Ranjha L, Hugener J, Adam C, Acharya A, Weyland N, Aran-Guiu X, Charbonnier J-B, et al Regulation of the MLH1-MLH3 endonuclease in meiosis. Nature. 2020:586(7830):618–622. 10.1038/s41586-020-2592-2 32814904

[kiae165-B6] Colas I , Macaulay M, Higgins JD, Phillips D, Barakate A, Posch M, Armstrong SJ, Franklin FCH, Halpin C, Waugh R, et al A spontaneous mutation in MutL-Homolog 3 (HvMLH3) affects synapsis and crossover resolution in the barley desynaptic mutant *des10*. New Phytologist. 2016:212(3):693–707. 10.1111/nph.14061 27392293

[kiae165-B7] Couteau F , Belzile F, Horlow C, Grandjean O, Vezon D, Doutriaux MP. Random chromosome segregation without meiotic arrest in both male and female meiocytes of a *dmc1* mutant of Arabidopsis. Plant Cell. 1999:11(9):1623–1634. 10.1105/tpc.11.9.1623 10488231 PMC144309

[kiae165-B8] Dion E , Li LL, Jean M, Beizile F. An Arabidopsis *MLH1* mutant exhibits reproductive defects and reveals a dual role for this gene in mitotic recombination. Plant J. 2007:51(3):431–440. 10.1111/j.1365-313X.2007.03145.x 17559505

[kiae165-B9] Draeger T , Martin AC, Alabdullah AK, Pendle A, Rey MD, Shaw P, Moore G. *Dmc1* is a candidate for temperature tolerance during wheat meiosis. Theor Appl Genet. 2020:133(3):809–828. 10.1007/s00122-019-03508-9 31853574 PMC7021665

[kiae165-B10] Du HY , Zeng XR, Zhao M, Cui XP, Wang Q, Yang H, Cheng H, Yu DY. Efficient targeted mutagenesis in soybean by TALENs and CRISPR/Cas9. J Biotechnol. 2016:217:90–97. 10.1016/j.jbiotec.2015.11.005 26603121

[kiae165-B11] Earley KW , Haag JR, Pontes O, Opper K, Juehne T, Song KM, Pikaard CS. Gateway-compatible vectors for plant functional genomics and proteomics. Plant J. 2006:45(4):616–629. 10.1111/j.1365-313X.2005.02617.x 16441352

[kiae165-B12] Ellison AR , Lofing J, Bitter GA. Human *MutL* homolog (MLH1) function in DNA mismatch repair: a prospective screen for missense mutations in the ATPase domain. Nucleic Acids Res. 2004:32(18):5321–5338. 10.1093/nar/gkh855 15475387 PMC524276

[kiae165-B13] Fayos I , Mieulet D, Petit J, Meunier AC, Périn C, Nicolas A, Guiderdoni E. Engineering meiotic recombination pathways in rice. Plant Biotechnol J. 2019:17(11):2062–2077. 10.1111/pbi.13189 31199561 PMC6790369

[kiae165-B14] Feng XX , Yang SX, Tang KQ, Zhang YH, Leng JT, Ma JJ, Wang Q, Feng XZ. *GmPGL1*, a thiamine thiazole synthase, is required for the biosynthesis of thiamine in soybean. Front Plant Sci. 2019:10:1546. 10.3389/fpls.2019.01546 31824549 PMC6883718

[kiae165-B15] Findley SD , Cannon S, Varala K, Du JC, Ma JX, Hudson ME, Birchler JA, Stacey G. A fluorescence *in situ* hybridization system for karyotyping soybean. Genetics. 2010:185(3):727–744. 10.1534/genetics.109.113753 20421607 PMC2907198

[kiae165-B16] Gao J , Yang S, Cheng W, Fu Y, Leng J, Yuan X, Jiang N, Ma J, Feng X. GmILPA1, encoding an APC8-like protein, controls leaf petiole angle in soybean. Plant Physiol. 2017:174(2):1167–1176. 10.1104/pp.16.00074 28336772 PMC5462013

[kiae165-B17] Gonzalo A , Lucas M-O, Charpentier C, Sandmann G, Lloyd A, Jenczewski E. Reducing *MSH4* copy number prevents meiotic crossovers between non-homologous chromosomes in *Brassica napus*. Nat Commun. 2019:10(1):2354. 10.1038/s41467-019-10010-9 31142748 PMC6541637

[kiae165-B18] Gottlob-McHugh SG , Lévesque M, MacKenzie K, Olson M, Yarosh O, Johnson DA. Organization of the 5S rRNA genes in the soybean *Glycine max* (L.) Merrill and conservation of the 5S rDNA repeat structure in higher plants. Genome. 1990:33(4):486–494. 10.1139/g90-072 2227404

[kiae165-B19] Gray S , Cohen PE. Control of meiotic crossovers: from double-strand break formation to designation. Annu Rev Genet. 2016:50(1):175–210. 10.1146/annurev-genet-120215-035111 27648641 PMC5319444

[kiae165-B20] Henderson IR . Control of meiotic recombination frequency in plant genomes. Curr Opin Plant Biol. 2012:15(5):556–561. 10.1016/j.pbi.2012.09.002 23017241

[kiae165-B21] Higgins JD , Buckling EF, Franklin FCH, Jones GH. Expression and functional analysis of *AtMUS81* in Arabidopsis meiosis reveals a role in the second pathway of crossing-over. Plant J. 2008:54(1):152–162. 10.1111/j.1365-313X.2008.03403.x 18182028

[kiae165-B22] Huang J , Li X, Wang C, Wang Y. Evaluation of crossover number, distribution, and interference using cytological assays in Arabidopsis. Current Protocols. 2022:2(12):e599. 10.1002/cpz1.599 36468904

[kiae165-B23] Hunter N , Borts RH. Mlh1 is unique among mismatch repair proteins in its ability to promote crossing-over during meiosis. Genes Dev. 1997:11(12):1573–1582. 10.1101/gad.11.12.1573 9203583

[kiae165-B24] Jackson N , Sanchez-Moran E, Buckling E, Armstrong SJ, Jones GH, Franklin FCH. Reduced meiotic crossovers and delayed prophase I progression in *AtMLH3*-deficient *Arabidopsis*. EMBO J. 2006:25(6):1315–1323. 10.1038/sj.emboj.7600992 16467846 PMC1422170

[kiae165-B25] Jean M , Pelletier J, Hilpert M, Belzile F, Kunze R. Isolation and characterization of *AtMLH1*, a *MutL* homologue from *Arabidopsis thaliana*. Mol Gen Genet. 1999:262(4–5):633–642. 10.1007/s004380051126 10628846

[kiae165-B26] Jing J-L , Zhang T, Kao Y-H, Huang T-H, Wang C-JR, He Y. *ZmMTOPVIB* enables DNA double-strand break formation and bipolar spindle assembly during maize meiosis. Plant Physiol. 2020:184(4):1811–1822. 10.1104/pp.20.00933 33077613 PMC7723106

[kiae165-B27] Kato KK , Palmer RG. Molecular mapping of the male-sterile, female-sterile mutant gene (*st8*) in soybean. J Heredity. 2003:94(5):425–428. 10.1093/jhered/esg085 14557397

[kiae165-B28] Ku JC , Ronceret A, Golubovskaya I, Lee DH, Wang CT, Timofejeva L, Kao YH, Angoa AKG, Kremling K, Williams-Carrier R, et al Dynamic localization of SPO11-1 and conformational changes of meiotic axial elements during recombination initiation of maize meiosis. PLoS Genet. 2020:16(4):e1007881. 10.1371/journal.pgen.1007881 32310948 PMC7192515

[kiae165-B29] Kumar S , Stecher G, Tamura K. MEGA7: molecular evolutionary genetics analysis version 7.0 for bigger datasets. Mol Biol Evol. 2016:33(7):1870–1874. 10.1093/molbev/msw054 27004904 PMC8210823

[kiae165-B30] Kurzbauer M-T , Pradillo M, Kerzendorfer C, Sims J, Ladurner R, Oliver C, Janisiw MP, Mosiolek M, Schweizer D, Copenhaver GP, et al *Arabidopsis thaliana* FANCD2 promotes meiotic crossover formation. Plant Cell. 2018:30(2):415–428. 10.1105/tpc.17.00745 29352063 PMC5868695

[kiae165-B31] Lhuissier FG , Offenberg HH, Wittich PE, Vischer NO, Heyting C. The mismatch repair protein MLH1 marks a subset of strongly interfering crossovers in tomato. Plant Cell. 2007:19(3):862–876. 10.1105/tpc.106.049106 17337626 PMC1867368

[kiae165-B32] Li YF , Cheng ZK. Fluorescence in situ hybridization on rice chromosomes. Methods Mol Biol. 2016:1370:105–112. 10.1007/978-1-4939-3142-2_8 26659957

[kiae165-B33] Li H , Durbin R. Fast and accurate short read alignment with Burrows–Wheeler transform. Bioinformatics. 2009:25(14):1754–1760. 10.1093/bioinformatics/btp324 19451168 PMC2705234

[kiae165-B34] Li X , Yu M, Bolaños-Villegas P, Zhang J, Da N, Ma H, Wang Y. Fanconi anemia ortholog FANCM regulates meiotic crossover distribution in plants. Plant Physiol. 2021:186(1):344–360. 10.1093/plphys/kiab061 33576801 PMC8154078

[kiae165-B35] Lu JY , Wang CL, Wang HY, Zheng H, Bai WT, Lei DK, Tian YL, Xiao YJ, You SM, Wang QM, et al *OsMFS1*/*OsHOP2* complex participates in rice male and female development. Front Plant Sci. 2020:11:518. 10.3389/fpls.2020.00518 32499797 PMC7243175

[kiae165-B36] Luo Q , Tang D, Wang M, Luo WX, Zhang L, Qin BX, Shen Y, Wang KJ, Li YF, Cheng ZK. The role of OsMSH5 in crossover formation during rice meiosis. Mol Plant. 2013:6(3):729–742. 10.1093/mp/sss145 23220939

[kiae165-B37] Mao B , Zheng W, Huang Z, Peng Y, Shao Y, Liu C, Tang L, Hu Y, Li Y, Hu L, et al Rice MutLγ, the MLH1-MLH3 heterodimer, participates in the formation of type I crossovers and regulation of embryo sac fertility. Plant Biotechnol J. 2021:19(7):1443–1455. 10.1111/pbi.13563 33544956 PMC8313138

[kiae165-B38] Mercier R , Mézard C, Jenczewski E, Macaisne N, Grelon M. The molecular biology of meiosis in plants. Annu Rev Plant Biol. 2015:66(1):297–327. 10.1146/annurev-arplant-050213-035923 25494464

[kiae165-B39] Moran ES , Armstrong SJ, Santos JL, Franklin FCH, Jones GH. Chiasma formation in *Arabidopsis thaliana* accession Wassileskija and in two meiotic mutants. Chromosome Res. 2001:9(2):121–128. 10.1023/A:1009278902994 11321367

[kiae165-B40] Neff MM , Turk E, Kalishman M. Web-based primer design for single nucleotide polymorphism analysis. Trends Genet. 2002:18(12):613–615. 10.1016/S0168-9525(02)02820-2 12446140

[kiae165-B41] Owen FV . A sterile character in soybeans. Plant Physiol. 1928:3(2):223–226. 10.1104/pp.3.2.223 16652565 PMC440003

[kiae165-B42] Palmer RG . Desynaptic mutant in soybean. J Heredity. 1974:65(5):280–286. 10.1093/oxfordjournals.jhered.a108529

[kiae165-B43] Palmer RG , Horner HT. Genetics and cytology of a genic male-sterile, female-sterile mutant from a transposon-containing soybean population. J Heredity. 2000:91(5):378–383. 10.1093/jhered/91.5.378 10994704

[kiae165-B44] Palmer RG , Sandhu D, Curran K, Bhattacharyya MK. Molecular mapping of 36 soybean male-sterile, female-sterile mutants. Theor Appl Genet. 2008:117(5):711–719. 10.1007/s00122-008-0812-5 18592206

[kiae165-B45] Paz MM , Martinez JC, Kalvig AB, Fonger TM, Wang K. Improved cotyledonary node method using an alternative explant derived from mature seed for efficient *Agrobacterium*-mediated soybean transformation. Plant Cell Rep. 2006:25(3):206–213. 10.1007/s00299-005-0048-7 16249869

[kiae165-B46] Pochon G , Henry IM, Yang C, Lory N, Fernández-Jiménez N, Böwer F, Hu B, Carstens L, Tsai HT, Pradillo M, et al The Arabidopsis Hop1 homolog ASY1 mediates cross-over assurance and interference. PNAS Nexus. 2023:2(3):1–17. 10.1093/pnasnexus/pgac302 PMC10042279 36992817

[kiae165-B47] Prolla TA , Christie DM, Liskay RM. Dual requirement in yeast DNA mismatch repair for *MLH1* and *PMS1*, two homologs of the bacterial *mutL* gene. Mol Cell Biol. 1994:14(1):407–415. 10.1128/mcb.14.1.407-415.1994 8264608 PMC358390

[kiae165-B48] Reyes GX , Schmidt TT, Kolodner RD, Hombauer H. New insights into the mechanism of DNA mismatch repair. Chromosoma. 2015:124(4):443–462. 10.1007/s00412-015-0514-0 25862369 PMC4600670

[kiae165-B49] Saini R , Singh AK, Hyde GJ, Baskar R. Levels of heterochiasmy during *Arabidopsis* development as reported by fluorescent tagged lines. G3 (Bethesda). 2020:10(6):2103–2110. 10.1534/g3.120.401296 32321838 PMC7263686

[kiae165-B50] Schmutz J , Cannon SB, Schlueter J, Ma JX, Mitros T, Nelson W, Hyten DL, Song QJ, Thelen JJ, Cheng JL, et al Genome sequence of the palaeopolyploid soybean. Nature. 2010:463(7278):178–183. 10.1038/nature08670 20075913

[kiae165-B51] Speth B , Rogers JP, Boonyoo N, VanMeter AJ, Baumbach J, Ott A, Moore J, Cina T, Palmer R, Sandhu D. Molecular mapping of five soybean genes involved in male-sterility, female-sterility. Genome. 2015:58(4):143–149. 10.1139/gen-2015-0044 26213292

[kiae165-B52] Tang K , Yang S, Feng X, Wu T, Leng J, Zhou H, Zhang Y, Yu H, Gao J, Ma J, et al *GmNAP1* is essential for trichome and leaf epidermal cell development in soybean. Plant Mol Biol. 2020:103(6):609–621. 10.1007/s11103-020-01013-y 32415514 PMC7385028

[kiae165-B53] Vimal D , Kumar S, Pandey A, Sharma D, Sainia S, Gupta S, Ram KR, Chowdhuri DK. Mlh1 is required for female fertility in *Drosophila melanogaster*: an outcome of effects on meiotic crossing over, ovarian follicles and egg activation. Eur J Cell Biol. 2018:97(2):75–89. 10.1016/j.ejcb.2017.12.002 29290392

[kiae165-B54] Wang Y , Cheng Z, Lu P, Timofejeva L, Ma H. Molecular cell biology of male meiotic chromosomes and isolation of male meiocytes in *Arabidopsis thaliana*. Methods Mol Biol. 2014:1110:217–230. 10.1007/978-1-4614-9408-9_10 24395259

[kiae165-B55] Wang Y , Copenhaver GP. Meiotic recombination: mixing it up in plants. Annu Rev Plant Biol. 2018:69(1):577–609. 10.1146/annurev-arplant-042817-040431 29489392

[kiae165-B56] Wang DM , Liang XX, Bao YZ, Yang SX, Zhang X, Yu H, Zhang Q, Xu GY, Feng XZ, Dou DL. A malectin-like receptor kinase regulates cell death and pattern-triggered immunity in soybean. EMBO Rep. 2020:21(11):e50442. 10.15252/embr.202050442 32924279 PMC7645207

[kiae165-B57] Wang YP , Tang HB, DeBarry JD, Tan X, Li JP, Wang XY, Lee TH, Jin HZ, Marler B, Guo H, et al MCScanx: a toolkit for detection and evolutionary analysis of gene synteny and collinearity. Nucleic Acids Res. 2012b:40(7):e49. 10.1093/nar/gkr1293 22217600 PMC3326336

[kiae165-B58] Wang CL , Wang Y, Cheng ZJ, Zhao ZG, Chen J, Sheng PK, Yu Y, Ma WW, Duan EC, Wu FQ, et al The role of OsMSH4 in male and female gamete development in rice meiosis. J Exp Bot. 2016:67(5):1447–1459. 10.1093/jxb/erv540 26712826 PMC4762385

[kiae165-B59] Wang KJ , Wang M, Tang D, Shen Y, Miao CB, Hu Q, Lu TG, Cheng ZK. The role of rice HEI10 in the formation of meiotic crossovers. PLoS Genet. 2012a:8(7):e1002809. 10.1371/journal.pgen.1002809 22792078 PMC3390396

[kiae165-B60] Xin XD , Li XW, Zhu JK, Liu XB, Chu ZH, Shen JL, Wu CY. OsMLH1 interacts with OsMLH3 to regulate synapsis and interference-sensitive crossover formation during meiosis in rice. J Genet Genomics. 2021:48(6):485–496. 10.1016/j.jgg.2021.04.011 34257043

[kiae165-B61] Yun J , Kim YS, Jung JH, Seo PJ, Park CM. The AT-hook motif-containing protein AHL22 regulates flowering initiation by modifying *FLOWERING LOCUS T* chromatin in Arabidopsis. J Biol Chem. 2012:287(19):15307–15316. 10.1074/jbc.M111.318477 22442143 PMC3346147

[kiae165-B62] Zhang M , Liu S, Wang Z, Yuan Y, Zhang Z, Liang Q, Yang X, Duan Z, Liu Y, Kong F, et al Progress in soybean functional genomics over the past decade. Plant Biotechnol J. 2022:20(2):256–282. 10.1111/pbi.13682 34388296 PMC8753368

[kiae165-B63] Zhang JP , Wang XZ, Lu YM, Bhusal SJ, Song QJ, Cregan PB, Yen Y, Brown M, Jiang GL. Genome-wide scan for seed composition provides insights into soybean quality improvement and the impacts of domestication and breeding. Mol Plant. 2018:11(3):460–472. 10.1016/j.molp.2017.12.016 29305230

[kiae165-B64] Zhou Z , Bi G, Zhou JM. Luciferase complementation assay for protein–protein interactions in plants. Curr Protoc Plant Biol. 2018:3(1): 42–50 10.1002/cppb.20066 30040251

[kiae165-B65] Zhou H , Tang K, Li G, Liu W, Yu H, Yuan X, Yang S, Bhattacharyya MK, Feng X. A robust and rapid candidate gene mapping pipeline based on M_2_ populations. Front Plant Sci. 2021:12:681816. 10.3389/fpls.2021.681816 34149782 PMC8207192

