# Supplementary material for: MutL homolog 1 participates in interference-sensitive meiotic crossover formation in soybean
Source: Plant Physiol. 2024 Mar 16;195(4):2579–95. doi: 10.1093/plphys/kiae165 (PMC11288737; doi:10.1093/plphys/kiae165)
Supplement: kiae165_Supplementary_Data [file kiae165_supplementary_data.pdf]

Supplemental Data

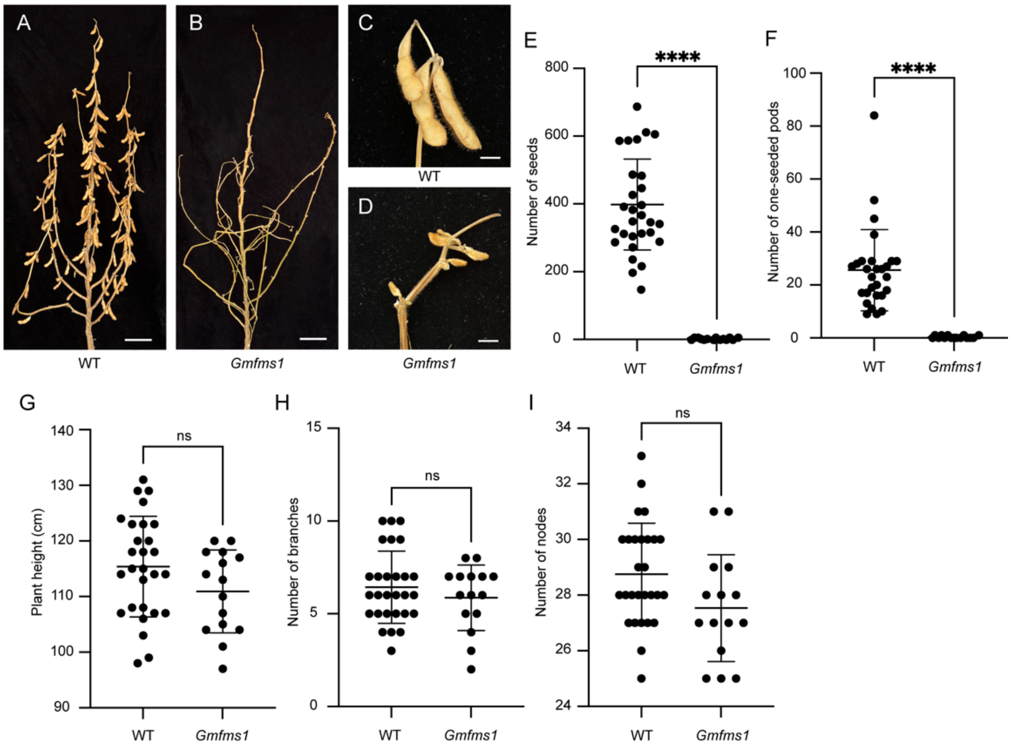

Supplemental Figure S1 Phenotypic assessment of the *Gmfms1* mutant at the full maturity stage. Plants of wild type (WT, A) and *Gmfms1* mutant (B). Scale bars = 10 cm. Pods of wild type (C) and *Gmfms1* mutant (D). Scale bars = 1 cm. (E–I) Statistical analyses of number of seeds (E), number of one-seeded pods (F), plant height (G), number of branches (H), and number of nodes (I) in the wild type (n = 28) and the *Gmfms1* mutant (n = 15). Asterisks indicate statistically significant differences (****$P < 0.0001$, Student's unpaired t-test); ns, not significant. All data shown are means ± SDs.

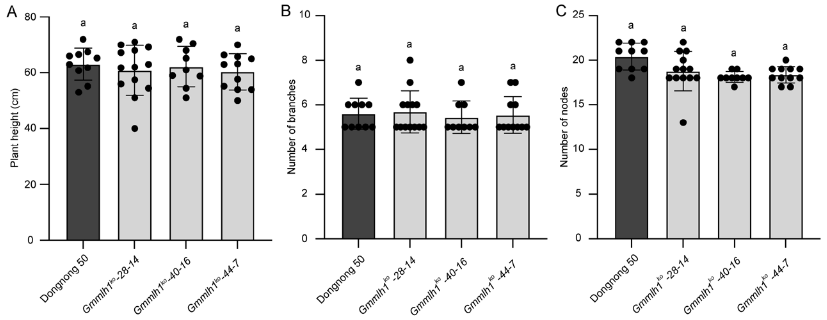

Supplemental Figure S2 Statistical analysis of the phenotypes of the *Gmmlh1* knock-out mutants. Statistical analysis of plant height (A), number of branches (B) and number of nodes (C) among Dongnong 50 (n = 10), *Gmmlh1*^{ko}*-28-14* (n = 13), *Gmmlh1*^{ko}*-40-16* (n = 9), and *Gmmlh1*^{ko}*-44-7* (n = 11). One-way analysis of variance (ANOVA) followed by a post-hoc Tukey's honestly significant difference (Tukey's HSD) test was performed. The α for statistical significance was set to 0.05. The lowercase letters indicate significant differences among means. All data shown are means ± SDs.

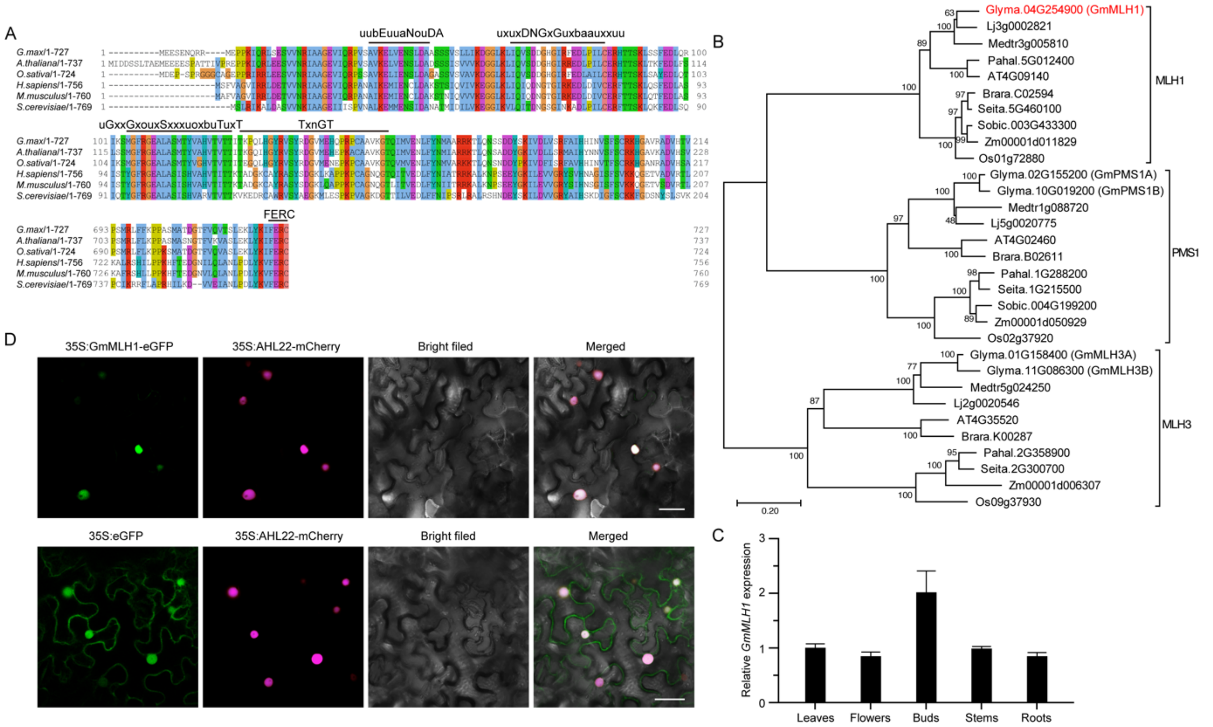

Supplemental Figure S3 Characterization of GmMLH1 protein. (A) Alignment of homologs MLH1 sequences from *Glycine max* (*G. max*), *Arabidopsis thaliana* (*A.thaliana*), *Oryza sativa* (*O. sativa*), *Homo sapiens* (*H. sapiens*), *Mus musculus* (*M. musculus*), and *Saccharomyces cerevisiae* (*S. cerevisiae*). The alignment was constructed using Clustal W. (B) Neighbor-joining phylogenetic tree of MutL homologs (MLH1, MLH3 and PMS1) from different species, including *Glycine max*, *Lotus japonicus*, *Medicago truncatula*, *Arabidopsis thaliana*, *Brassica rapa*, *Panicum hallii*, *Setaria italica*, *Sorghum bicolor*, *Zea mays*, and *Oryza sativa*. A neighbor-joining phylogenetic tree were constructed using MEGA 7. The bootstrap value was set at 1,000 replicates. (C) Relative expression of *GmMLH1* in different tissues of the wild type (Williams 82). Values are means ± SDs of three biological replicates normalized to expression of *GmMLH1* in the leaf sample, which was set to 1.0. (D) Subcellular localization of the GmMLH1-eGFP fusion protein in leaf epidermal cells of *Nicotiana benthamiana*. AHL22-mCherry was used as the nuclear marker, free eGFP was used as control. Scale bars = 50 µm.

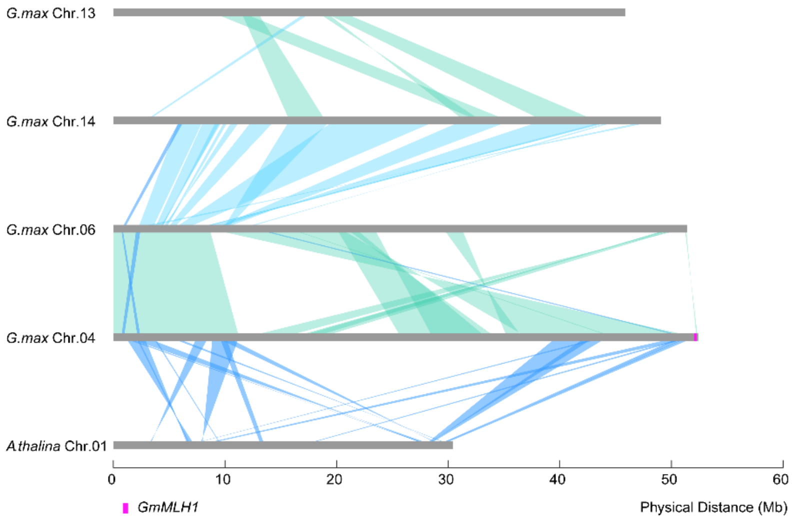

Supplemental Figure S4 Synteny plot of chromosomes 4, 6, 13, and 14 in soybean. The gray lines correspond to the chromosomes, while the magenta box represents the *GmMLH1* gene. The green region indicates the duplication event, which occurred during approximately 13 million years ago, the light blue region indicates the duplication event, which occurred approximately 53 million years ago, and the blue region indicates the duplication event, which occurred approximately 110 million years ago. *G. max* indicates *Glycine max*, *A. thaliana* indicates *Arabidopsis thaliana*, and Chr. indicates Chromosome.

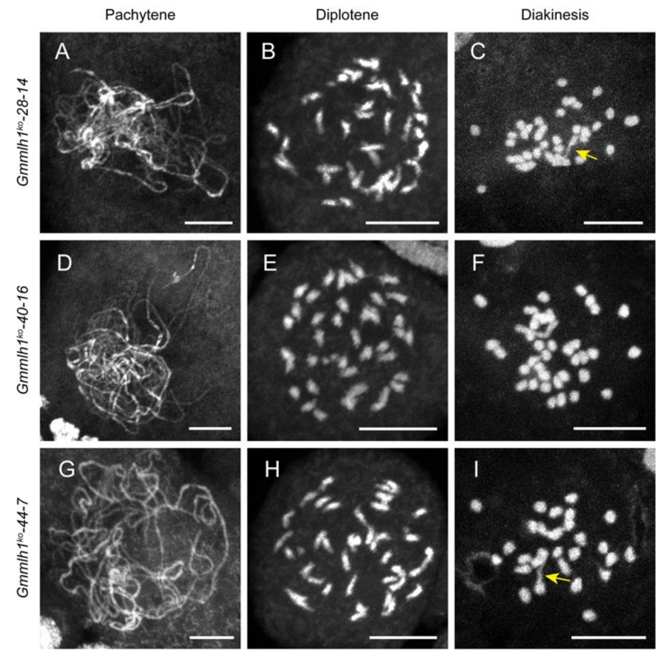

Supplemental Figure S5 Meiotic chromosome behaviors in the pollen mother cells from the *Gmmlh1* knock-out mutants. The *Gmmlh1* knockout mutant from *Gmmlh1^{ko}-28-14* line (A–C), *Gmmlh1^{ko}-40-16* line (D–F), the *Gmmlh1^{ko}-44-7* line (G–I). (A, D, G) Pachytene. (B, E, H) Diplotene. (C, F, I) Diakinesis. Arrows indicate bivalents. Scale bars = 10 μm.

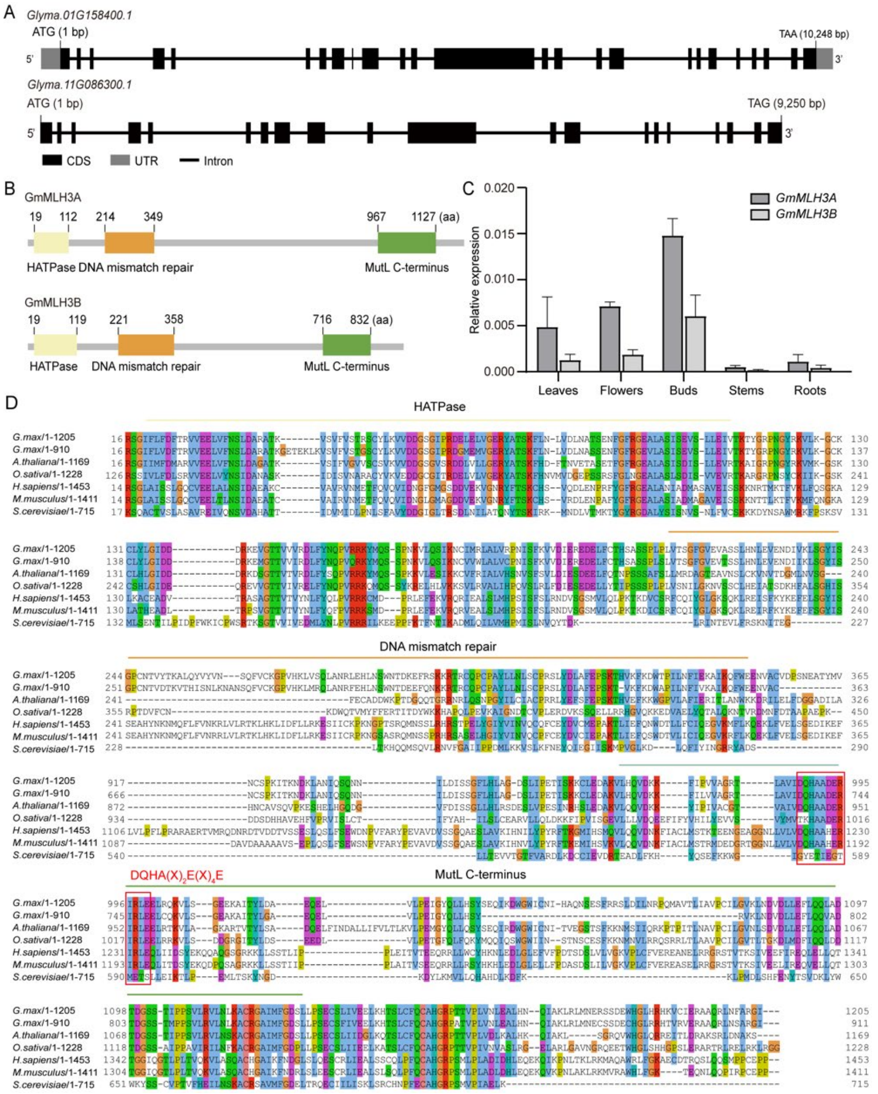

Supplemental Figure S6 Characterization of the GmMLH3 in soybean. (A) The structures of the *Glyma.01G158400.1* (*GmMLH3A*), and *Glyma.11G086300.1* (*GmMLH3B*) gene. The gray boxes indicate untranslated regions (UTR) and the black boxes indicate coding sequences (CDS). (B) The structures of the GmMLH3A, and GmMLH3B proteins in soybean. The boxes from left to right demarcate HATPase (GHKL) domains, DNA mismatch repair domains, and MutL C-terminus domain, respectively. (C) Relative expression patterns of *GmMLH3A* and *GmMLH3B* in soybean tissues. Values shown are means ± SDs of three biological replicates. (D) Alignment of homologous predicted MLH3 sequences from *Glycine max* (*G. max*), *Arabidopsis thaliana* (*A. thaliana*), *Oryza sativa* (*O. sativa*), *Homo sapiens* (*H. sapiens*), *Mus musculus* (*M. musculus*), and *Saccharomyces cerevisiae* (*S. cerevisiae*). The alignment was constructed using Clustal W. The metal binding motif DQHA(X)$_2$E(X)$_4$E within the MutL C-terminus domain is indicated in red box.

Supplemental Table S1. The reciprocal cross tests of Williams 82 and *Gmfms1* mutant.

Groups	Williams 82 ♂ × Williams 82♀	Williams 82 ♂ × *Gmfms1* ♀	*Gmfms1* ♂ × Williams 82 ♀
Pod-setting rate	53.66% (n = 41)	1.27% (n = 263)	14.29% (n = 42)

Supplemental Table S2. CentGm and 5S rDNA probe sequences in soybean.

Probe name	Sequence (5’→3’)
CentGm	TTCAATTTCGAGCGTCTCGATATATTA
5S rDNA	AGGTGCGATCATACCAGCACTAATGCACCGGATCCCATCAGAACTCCGC<br>AGTTAAGCGTGCTTGGGCGAGAGTAGTACTAGGATGGGTGACCTCCTGG<br>GAAGTCCTCGTGTTGCACCTCTTTTTACGTTTTTTTTTTTCTTTTTGCCCTT<br>ATTCTGAGTATTTTTCTTTGAAGCGAAGTAAAAGGTCCGATAAGTTAACT<br>AATTTTTGTGATTGATCGGGAGATAAATGTATCGTGGGCGCCGTGGCTCG<br>TTCGGTGTAGAAAGTCGATCGAAAGTCGGTCCGTCCGGGCAGGCAGAAG<br>GAATATAGTAATTGATTGTGCAATACTTATC

Supplemental Table S3. Primers used in this study.

**Primer name**	**Sequence (5'→3')**	**Description**
MOL0575-F	CTGTCACTCTATATCACACT	*GmMLH1* gene mapping
MOL0575-R	GAAATTATATTCCTACCTACGG	*GmMLH1* gene mapping
MOL0717-F	GTCCGAGGCAGCAGGAGAGGAC	*GmMLH1* gene mapping
MOL0717-R	GGCGCTGTCTGGATCTTCCA	*GmMLH1* gene mapping
MOL1277-F	GTCGATCGCTCGCGATTCCCT	*GmMLH1* gene mapping
MOL1277-R	CTTCATCATCGCTAGGCTCTAC	*GmMLH1* gene mapping
MOL9624-F	GGTATGTCTTCAAACATAAG	*GmMLH1* gene mapping
MOL9624-R	CGTCTATGGATTGGATATTC	*GmMLH1* gene mapping
MOL9626-F	TAAGCGAGGCATATGCGCTT	*GmMLH1* gene mapping
MOL9626-R	AGAGATAAACGACTCGTTGG	*GmMLH1* gene mapping
Gmmlh1-CAPS-F	CTCAGCCCTATGCCAACAGGTAT	CAPS marker for *Gmmlh1* genotype
Gmmlh1-CAPS-R	CAAAGTAGTTTGTCTGCTTCAAAT	CAPS marker for *Gmmlh1* genotype
GmMLH1-CDS-F	GAGCCGAATTGAGAAGTTGTGTATG	*GmMLH1* CDS cloning
GmMLH1-CDS-R	CCCAAACCAACGCAAAAACTGAT	*GmMLH1* CDS cloning
5S rDNA-F	AGGTGCGATCATACCAGCACT	5S rDNA cloning and probe labeling
5S rDNA-R	GATAAGTATTGCACAATCAATTAC	5S rDNA cloning and probe labeling
GmMLH1-CR-F	ATTGTGGTGAACCGAATCGCCGC	*GmMLH1* CRISPR/Cas9 gene editing
GmMLH1-CR-R	AACGCGGCGATTCGGTTCACCAC	*GmMLH1* CRISPR/Cas9 gene editing
Gmmlh1-CR-seq-F	CTGTGAGCTGAGGACAAATTTAACC	*GmMLH1* CRISPR/Cas9 target site detection

Gmmlh1-CR-seq-R	CCATTATCTGCGTTCCTTTAACAGC	*GmMLH1* CRISPR/Cas9 target site detection
GmMLH1-qPCR-F	TTAAGTCGATGGGCTTTCGC	qPCR for *GmMLH1*
GmMLH1-qPCR-R	CAACCATTATCTGCGTTCCTTT	qPCR for *GmMLH1*
GmMLH3A-qPCR-F	TGAGAACTTCGGTTTTCGTGG	qPCR for *GmMLH3A*
GmMLH3A-qPCR-R	TGGTGCCCACTTCCTTTCTAT	qPCR for *GmMLH3A*
GmMLH3B-qPCR-F	ATGGGATGGAAATGGTGGGAG	qPCR for *GmMLH3B*
GmMLH3B-qPCR-R	CGCGAAAACCGAAGTTCTCAC	qPCR for *GmMLH3B*
GmMLH1-sl-F	CGGTACCCGGGGATCATGGAAGAGAGCGAGAATCAG	GmMLH1 subcellular localization assay
GmMLH1-sl-R	CGACTCTAGAGGATCGCATCTTTCAAAAATCTTATACAGT	GmMLH1 subcellular localization assay
GmMLH1-BiFC-F	TTGACTTTGCGGATCCATGGAAGAGAGCGAGAATCAG	BiFC assay for *GmMLH1*
GmMLH1-BiFC-R	GAGACTTTCGGGATCCGCATCTTTCAAAAATCTTATACAGT	BiFC assay for *GmMLH1*
GmMLH3A-BiFC-F	TTGACTTTGCGGATCCATGGCCAGCATAAAGCCTTTGC	BiFC assay for *GmMLH3A*
GmMLH3A-BiFC-R	GAGACTTTCGGGATCCAATTCCTCTAGCAAAATTTAAACGC	BiFC assay for *GmMLH3A*
GmMLH3B-BiFC-F	TTGACTTTGCGGATCCATGGCGAGCATAAAGTTATTGTCGG	BiFC assay for *GmMLH3B*
GmMLH3B-BiFC-R	GAGACTTTCGGGATCCAATTCCTCTAGCAGAATTTAAACGC	BiFC assay for *GmMLH3B*
GmMLH1-Luc-F	ACGGGGGACGAGCTCGGTACCATGGAAGAGAGCGAGAATCAGAGGC	Split-LUC assay for *GmMLH1*
GmMLH1-Luc-R	AACATCGTATGGGTAGTCGACGCATCTTTCAAAAATCTTATACAGT	Split-LUC assay for *GmMLH1*
GmMLH3A-Luc-F	ACGGGGGACGAGCTCGGTACCATGGCCAGCATAAAGCCTTTGCCGG	Split-LUC assay for *GmMLH3A*
GmMLH3A-Luc-R	CGCGTACGAGATCTGGTCGACAATTCCTCTAGCAAAATTTAAACGC	Split-LUC assay for *GmMLH3A*
GmMLH3B-Luc-F	ACGGGGGACGAGCTCGGTACCATGGCGAGCATAAAGTTATTGTCGG	Split-LUC assay for *GmMLH3B*
GmMLH3B-Luc-R	CGCGTACGAGATCTGGTCGACAATTCCTCTAGCAGAATTTAAACGC	Split-LUC assay for *GmMLH3B*
GmMLH1-pd-F	GCGTGGATCCCCGAATTCATGGAAGAGAGCGAGAAT	Pull-down assay for *GmMLH1*

GmMLH1-pd-R	CGATGCGGCCGCTCGAGTTAGCATCTTTCAAAAATC	Pull-down assay for *GmMLH1*
GmMLH3A-pd-F	ATCGAAGGTAGGCATATGATGGCCAGCATAAAGCCT	Pull-down assay for *GmMLH3A*
GmMLH3A-pd-R	GTCGACAAGCTTGAATTCTCAAATTCCTCTAGCAAA	Pull-down assay for *GmMLH3A*
GmMLH3B-pd-F	ATCGAAGGTAGGCATATGATGGCGAGCATAAAGTTATTGTCGG	Pull-down assay for *GmMLH3B*
GmMLH3B-pd-R	GGTCGACAAGCTTGAATTCTCAAATTCCTCTAGCAGAATTTAAACGC	Pull-down assay for *GmMLH3B*
